# Response of *Rhodococcus cerastii* IEGM 1278 to toxic effects of ibuprofen

**DOI:** 10.1371/journal.pone.0260032

**Published:** 2021-11-18

**Authors:** Irina B. Ivshina, Elena A. Tyumina, Grigory A. Bazhutin, Elena V. Vikhareva

**Affiliations:** Perm Federal Research Center of the Ural Branch of the Russian Academy of Sciences, Perm, Russia; Carnegie Mellon University, UNITED STATES

## Abstract

The article expands our knowledge on the variety of biodegraders of ibuprofen, one of the most frequently detected non-steroidal anti-inflammatory drugs in the environment. We studied the dynamics of ibuprofen decomposition and its relationship with the physiological status of bacteria and with additional carbon and energy sources. The involvement of cytoplasmic enzymes in ibuprofen biodegradation was confirmed. Within the tested actinobacteria, *Rhodococcus cerastii* IEGM 1278 was capable of complete oxidation of 100 μg/L and 100 mg/L of ibuprofen in 30 h and 144 h, respectively, in the presence of an alternative carbon source (*n*-hexadecane). Besides, the presence of ibuprofen induced a transition of rhodococci from single- to multicellular lifeforms, a shift to more negative zeta potential values, and a decrease in the membrane permeability. The initial steps of ibuprofen biotransformation by *R*. *cerastii* IEGM 1278 involved the formation of hydroxylated and decarboxylated derivatives with higher phytotoxicity than the parent compound (ibuprofen). The data obtained indicate potential threats of this pharmaceutical pollutant and its metabolites to biota and natural ecosystems.

## Introduction

The intensively developing pharmaceutical industry and the growing uncontrolled consumption of drugs in human and veterinary medicine have formed a new type of hazardous emerging contaminants. These include a large group of substances collectively termed “pharmaceutical pollutants”. Pharmaceutical pollutants, which are highly stable compounds with a diverse chemical nature and pronounced bioactivities, have been recognized as a new class of xenobiotics since the early 2000s [1, 2]. In terms of scale and environmental significance, the problem of pharmaceutical pollution is now becoming genuinely planetary [3–5]. Pharmaceutical pollutants have harmful effects on the environment, causing high toxicity even in environmentally relevant concentrations [6–8]. Understanding the mechanisms of biological transformation of pharmaceuticals is essential for determining their ecological fate and approaches for effective neutralization. It is impossible to assess the ecological risks posed by these micropollutants without studying the nature and persistence of metabolites formed during their transformation. Fundamental knowledge is needed about the possibility of their biodegradation by microorganisms in polluted environments, which act as a primary response system to potentially dangerous changes in their habitats and trigger the mechanisms of detoxification and decomposition of xenobiotics at the earliest stage.

Among the microorganisms, which carry out the processes of natural attenuation of anthropogenic xenobiotics are actinobacteria, typical inhabitants of aquatic and soil ecosystems, with the greatest variety of degradable pollutants and a wide range of adaptive capabilities [9–11]. Effective actinobacterial degradation of selected antibiotics, hormones, antiepileptics, analgesics, and nonsteroidal anti-inflammatory drugs (NSAIDs) has already been documented [12–17]. Earlier, we confirmed the ability of rhodococci to completely biodegrade pharmaceuticals from analgesics and antispasmodics, including paracetamol [18], drotaverine hydrochloride [19, 20], and diclofenac sodium [21, 22]. In this work, the analysis of possible *Rhodococcus* spp. participation as biooxidants of ibuprofen (IBP), a monocyclic NSAID and a propionic acid derivative most often detected in the environment was of interest.

IBP is a popular drug in human and veterinary medicine and is included in the WHO “Essential Drug List”; it has anti-inflammatory, antipyretic, and analgesic effects. It is used to treat osteoarthritis, gout, pericarditis, and cancer [23, 24]. IBP production volumes are estimated at thousands of tons per year [25]. IBP is released into the environment mostly through wastewater due to its widespread usage, highly stable molecule, and incomplete breakdown in the human body, as well as the improper disposal practice of unused and expired IBP [24, 26, 27]. IBP is ubiquitous in surface, ground, and treated wastewater in concentrations ranging from a few ng/L to 6,000 μg/L and is also regularly detected in drinking water [2, 24, 28–34].

The high (log K_ow_ 3.49) lipophilicity of IBP due to the presence of a 2-methylpropyl radical and absence of additional oxygen atoms determines its ability to penetrate biological membranes and a high (0.18 l/kg) degree of distribution in living organisms [35]. In this regard, IBP is prone to bioaccumulate in marine [36, 37] and freshwater [38–40] molluscs, fish [41, 42], mammals [43], plants [44] and to biomagnificate in food chains [38, 45]. Accumulating in the body of vertebrates and invertebrates, IBP causes negative effects, such as oxidative stress, DNA damage, suppression of individual enzyme activities (e.g., nitration of proteins), disruption of mitochondria, and lipid peroxidation [6, 16, 46–49]. The toxicity of IBP to lower and higher plants is described in a few studies [48, 50]. At the same time, the majority of ecotoxicological studies are devoted only to IBP itself, while information on detection and toxicity of its partial oxidation products is quite scarce [51–53]. There are only a few data indicating negative effects of IBP and its metabolites, other NSAIDs included, on nitrogen fixation, induction of oxidative stress in microorganisms, and destabilization of cell membranes [21, 54, 55]. IBP has recently been shown to facilitate the spreading of antibiotic resistance through the uptake of exogenous antibiotic resistance genes [56]. The underlying mechanisms of this phenomenon were affiliated with IBP-induced bacterial competence, oxidative stress accompanied with over-production of reactive oxygen species, and increase in cell membrane permeability.

The aim of this study was to evaluate the ability of actinobacteria to bioconvert IBP and to investigate the mechanisms of the pharmaceutical’s impacts on bacterial cells. In this work, we studied the relation between IBP biotransformation and the physiological states and cultivation conditions of actinobacteria, as well as the influence of IBP on the response of the bacterial cells. Metabolites formed at the initial stages of IBP biooxidation were detected, and possible reactions of bacterial transformation of IBP were described. This is the first report on the ability of *Rhodococcus* spp. to degrade IBP.

## Materials and methods

### Bacterial strains

In this work, 100 strains of actinobacteria from the Regional Specialised Collection of Alkanotrophic Microorganisms (acronym IEGM, the World Federation for Culture Collections # 285, http://www.iegmcol.ru), belonging to the genera *Agromyces* (1 strain), *Brachybacterium* (2 strains), *Clavibacter* (1 strain), *Corynebacterium* (1 strain), *Curtobacterium* (1 strain), *Dermacoccus* (1 strain), *Dietzia* (15 strains), *Gordonia* (7 strains), *Micrococcus* (2 strains), *Microbacterium* (1 strain), *Nocardioides* (2 strains), *Rhodococcus* (65 strains), and *Williamsia* (1 strain) were employed. The strains were selected by geography and isolation sources and also by the well-known catalytic activity to complex hydrophobic organic compounds.

### Reagents

IBP (C_13_H_17_O_2_Na; CAS: 31121-93-4; (RS)-2-(4-(2-methylpropyl) phenyl)propanoic acid in the form of sodium salt) was used as a pharmaceutical substance (colourless crystalline powder with a characteristic odor, 98.0% purity, moderately soluble in water) produced by Sigma-Aldrich, USA. Chemicals, including acetonitrile, ethyl acetate, and ethanol, were of chemical, analytical, or extra-pure grades (Cryochrome, Russia; Merck, Germany; Sigma-Aldrich, USA). Millipore Simplicity Personal Ultrapure Water System (Millipore, USA) was used to obtain ultrapure water for high-performance liquid chromatography.

### Minimum inhibitory concentration of IBP

Minimum inhibitory concentrations (MIC) of IBP were determined by the microplate method, followed by cell staining with 0.2% solution of iodonitrotetrazolium chloride (Sigma-Aldrich, USA) [21]. The cell viability was assessed by measuring the optical density (OD_630_) of the stained culture using a Multiscan Ascent microplate spectrophotometer (Thermo Electron Corporation, USA). MIC tests were carried out in eight replicates.

### IBP biotransformation

In experiments on IBP biotransformation, a mineral salt medium RS (g/L): K_2_HPO_4_−2.0; KH_2_PO_4_−2.0; KNO_3_−1.0; (NH_4_)_2_SO_4_−2.0; NaCl– 1.0; MgSO_4_ × 7 H_2_O – 0.2; CaCl_2_ × 2 H_2_O – 0.02; FeCl_3_ × 7 H_2_O – 0.001 (pH 6.9) supplemented with a trace element solution was used [57]. IBP was added into the mineral medium as a sterile concentrated aqueous solution (1,000 mg/L) to a final concentration of 100 mg/L or 100 μg/L. As an additional source of carbon and energy, 10 different cosubstrates were tested: sodium acetate, glucose, oleanolic, phenylacetic and humic acids (0.1%); nutrient broth (NB, 1.3%); glycerol, pentanol-1, hexanol-1, and *n*-hexadecane (0.1 vol. %). Actinobacteria pre-grown for 1, 2, 3, or 4 days in NB (Oxoid, UK) and washed twice with a phosphate buffer (pH 7.0) were used as inocula (1 mL of OD_600_ 1.0). IBP biotransformation experiments were carried out at 160 rpm, 28°C in Erlenmeyer flasks with a capacity of 250 mL and a medium volume of 100 mL. Scaling of the IBP biotransformation process was performed in a BioFlo/CelliGen 115 stirred bioreactor (Eppendorf, New Brunswick, USA) with 4.0 L of medium, at 160 rpm and 28°C aerated with atmospheric air through a ring sparger at a constant rate of 0.3 L/min.

As controls, (a) sterile IBP solution in the mineral salt medium (to assess the abiotic degradation of IBP); (b) sterile IBP solution in the mineral salt medium with inactivated bacterial cells (to assess the degree of IBP adsorption on bacterial cells); (c) mineral salt medium containing *n*-hexadecane with bacterial cells without IBP (control for the differentiation of metabolites resulting from the decomposition of IBP) were used.

### Preparation of cell extracts

Crude cell extracts were obtained according to the method described by Tarasova et al. [58]. Bacteria pre-grown for two days in NB in the presence of IBP (10 mg/L) were washed three times and resuspended in a phosphate buffer (pH 7.0). The cell suspension was treated with a Soniprep 150 ultrasonic homogenizer (MSE, UK) for 60 min at the amplitude of 15 μm under cooling conditions. After sonication, no more than 0.1% of the cells survived, as confirmed by the calculation of CFU/mL and microscopic studies. The resulting homogenate was centrifuged (6,000 rpm, 20 min, 4°C) to obtain a fraction of cytoplasmic enzymes. To isolate the membrane-bound enzymes, the precipitate was resuspended in a 1% Triton X-100 solution (Sigma-Aldrich, USA) in a phosphate buffer (pH 7.0), stirred in an orbital shaker for 30 min, and then centrifuged (6,000 rpm, 20 min, 4°C). The residue containing the non-extractable enzymes was resuspended in a phosphate buffer (pH 7.0). The whole-cell complex in a phosphate buffer solution (pH 7.0) was used as a control.

### Microscopic studies

Cells were visualized using an Axio Imager M2 optical microscope (Zeiss, Germany) in phase contrast and fluorescence mode. Photo documentation of the images was carried out using the Axoicam 506 Color camera and Zen Blue 3.1 (Zeiss, Germany). The effect of IBP on cell surface morphology and relief was studied using a combined scanning system consisting of an MFP-3D-BIO^TM^ atomic force microscope (AFM) (Asylum Research Inc., USA) and an Olympus Fluo View 1000 confocal laser microscope (CLSM) (Olympus Corporation, Japan). The differentiation between live and dead cells was performed with a LIVE/DEAD^®^ *Bac*Light^TM^ Bacterial Viability Kit (Molecular Probes, USA). Preparation and scanning of samples were carried out following the previously described method by Ivshina et al. [21]. Root-mean-square roughness, length and width of cells were calculated from the height images. Cell volume and surface area were calculated using equations for cylindrical bodies [59]. The obtained images were processed using Igor Pro 6.22A (WaveMetrics, USA).

### Respirometry

The respiratory activity of cells was evaluated using a 10-channel Micro-Oxymax^®^ respirometer (Columbus Instruments, USA). The experiments were carried out in Micro-Oxymax glass flasks with 100 mL of the mineral salt medium containing 0.1% *n*-hexadecane (biotic control) or 0.1% *n*-hexadecane and 100 mg/L IBP (160 rpm, 28±2°C). The amount (μL) and rate (μL/h) of O_2_ consumed and CO_2_ released were measured. The respiratory activity was registered automatically every 30 min for 7 days.

### Zeta potential measurements

Zeta potentials of bacterial cells were measured by dynamic light scattering using the ZetaSizer Nano ZS analyzer (Malvern Instruments, UK) with the Malvern ZetaSizer software, v. 2.2. Cells grown in the mineral salt medium in the presence of 100 mg/L IBP and 0.1% *n*-hexadecane or only 0.1% *n*-hexadecane (biotic control) were washed twice and resuspended in 0.1 M KNO_3_ (pH 7.0) until OD_600_ 0.2 was reached. The measurements were carried out in U-shaped cuvettes with gold-plated electrodes at 25°C and pH 7.0.

### Membrane permeability measurements

Changes in the permeability of cell membranes under the influence of IBP were determined by crystal violet assays [60]. Bacterial cells cultured in the presence of 100 mg/L IBP and 0.1% *n*-hexadecane or only 0.1% *n*-hexadecane (biotic control) were centrifuged at 9,000×g and 4°C for 5 min and resuspended in a phosphate buffer (pH 7.4) containing 10 μg/mL of crystal violet. The suspensions were incubated at 28°C for 10 min and then centrifuged at 13,400×g for 15 min. The optical density of the supernatant was measured using a Lambda EZ201 spectrophotometer (Perkin-Elmer, USA) at a wavelength of 520 nm. A CV solution was used as a control. The percentage of crystal violet (UCV) uptake by cells was calculated as:

UCV=ODvalueofsample/ODvalueofCVsolution×100.

All experiments (whole-cell and crude cell extract biotransformation, respirometry, zeta potential, and membrane permeability measurements) were performed in triplicate, and the data are presented as mean ± standard deviation.

### Analytical methods

The removal of IBP during biotransformation was monitored by high-performance liquid chromatography (HPLC) using an LC Prominence 20A chromatograph (Shimadzu, Japan) equipped with a reversed-phase column Phenomenex Jupiter^®^ 5u C18 300 A, 250×4.60 mm, 5 μm (Phenomenex, USA) and a diode-matrix detector (SPD-M20A). Optimal conditions for determining IBP: a mobile phase–phosphate buffer solution (pH 5.0)–acetonitrile (40:60), eluent flow– 0.5 mL/min, column temperature– 40°C, sample volume– 20 μl, and detection wavelength– 254 nm. The IBP retention time was 9.28±0.20 min. The chromatographic information was recorded and processed using the LCSolution software (v/1.25 rus). Chromatographic peaks were normalized by sample (external standart) to make the data comparable across samples.

Products of bacterial IBP metabolism were analyzed using an LCMS-8050 liquid triple quadrupole chromatograph (Shimadzu, Japan) coupled with a mass spectrometric detector with a dual ionization source (electrospray and chemical ionization at atmospheric pressure). Metabolites were separated on a reversed-phase column Luna 3u C18 (2) 100A, 100×0.5 mm (Phenomenex, USA). As a mobile phase, acetonitrile and 0.1% formic acid were used in a ratio of 60:40 in the isocratic elution mode; the eluent flow rate was 1 mL/min; the column temperature was 40°C; the volume of the injected sample was 10 μL; the reference wavelength for detecting the target compound was 220 nm.

For chromatographic analysis, an aliquot (1 mL) of the rhodococcal culture fluid was centrifuged at 10,000 rpm for 5 min. The supernatant was filtered through a membrane nylon syringe filter (Filter-Bio, China) with a pore diameter of 0.20 μm.

For infrared (IR) spectroscopy analysis of IBP and its metabolites, the rhodococcal culture medium was acidified with 10% aqueous HCl solution to pH 2.0 and extracted three times with an equal volume (10 ml) of chloroform. The combined extracts were dried with Na_2_SO_4_. The solvent was removed on a rotary vaporizer Laborota 4000 (Heidolph, Germany). The IR spectra of the dry residues obtained after evaporation of a mixture of IBP biodegradation products were measured in KBr tablets on a SPECORD M-80 IR spectrophotometer (Carl Zeiss Jena, Germany).

### Phytotoxicity of IBP and its biotransformation products

Phytotoxicity of IBP biotransformation products to oat *Avena sativa* L. was evaluated as previously reported by Synowiec et al. [61]. In the experiments, seeds (Permagrobusiness, Russia) with the germination rate of 98% were used. The seeds were germinated for 3 days in sterile Petri dishes with filter paper treated with distilled water (5 mL). The germinated seeds (n = 25) were treated with 5 mL of an aqueous solution of IBP (100 mg/L) or its biotransformation products. After a 7-day degradation experiment, the culture medium containing neither IBP nor *n*-hexadecane was filtered through a membrane filter (0.20 μm) to obtain the transformation products’ solution without bacterial cells. All Petri dishes were placed in a growth chamber at a constant temperature of 25°C for 7 days. Toxicity was determined by the inhibition effect on the growth of roots according to the formula:

$$E_{in}=\frac{L_{c}-L_{e}}{L_{c}}\cdot 100\%,$$

where *E*_*in*_ is the inhibition effect, %; *L*_*e*_ is the average root length in the experiment, cm; *L*_*c*_ is the average root length in the control, cm. The phytotoxic effect was considered proven if the phytoeffect (*E*_*in*_) was ≥ 20% [61]. Germinated oat seeds treated with sterile distilled water were used as a control. Phytotoxicity test was performed in triplicate.

### *In silico* studies of IBP and its biotransformation products

The ecotoxicity of IBP and its bioconversion products was assessed using the computerized QSAR tool ECOSAR (Ecological Structure Activity Relationships) available in the EPI Suite^TM^ (The Estimation Programs Interface, EPA, USA). ECOSAR provides an opportunity to estimate the potential acute and chronic toxicities of chemicals to aquatic and terrestrial organisms using a computerized analysis of the structural and functional relationship in molecules. Ecotoxicity results were predicted based on the data available on the toxic effects of different organic chemicals.

The biodegradability of IBP metabolites was evaluated using the BioWin program (EPI Suite, EPA, USA). BioWin evaluates the possibility of rapid aerobic and anaerobic biodegradation of organic compounds in the presence of a mixed microbial population. Estimations were made in BioWin 5 (linear model) and 6 (non-linear model) and BioWin 7 (anaerobic model) software packages. The models gave biodegradation results for each compound, meaning that when the values are greater or equal to 0.5, they correspond to high biodegradability of the compound and less than 0.5 corresponds to low biodegradability of the compound.

The ability of IBP and its biotransformation products to settle in soil, bioconcentrate and bioaccumulate in aquatic organisms (fish) was evaluated using the KOCWIN and BCFBAF programs (EPI Suite, EPA, USA), modeling the corresponding values based on the octanol/water distribution coefficient (log K_ow_).

## Results and discussion

### Determination of actinobacterial resistance to IBP

All the actinobacterial strains tested remained viable when exposed to IBP in 125 to 1,000 mg/L concentration range (Table 1).

**Table 1:** Minimal inhibitory concentration (MIC) of IBP obtained for the tested actinobacterial strains.

Family	Species	MIC, mg/L
*Corynebacteriaceae*	*Corynebacterium variabile*	≥1,000
*Dermabacteraceae*	*Brachybacterium faecium*, *B*. *paraconglomeratum*	
*Dermacoccaceae*	*Dermacoccus nishinomiyaensis*	
*Gordoniaceae*	*Gordonia terrae*	
*Microbacteriaceae*	*Agromyces mediolanus*, *Curtobacterium citreum*	
*Micrococcaceae*	*Micrococcus luteus*, *M*. *lylae*	
*Nocardiaceae*	*Rhodococcus cerastii*, *R*. *cercidiphylli*, *R*. *corynebacterioides*	
*Nocardioidaceae*	*Nocardioides albus*, *N*. *jensenii*	
*Nocardiaceae*	*Rhodococcus opacus*	500– ≥1,000
*Gordoniaceae*	*Gordonia rubripertincta*	500
*Nocardiaceae*	*Rhodococcus globerulus*	
*Nocardiaceae*	*Rhodococcus fascians*	250 – ≥1,000
*Gordoniaceae*	*Williamsia marianensis*	250
*Micrococcaceae*	*Microbacterium imperiale*	
*Nocardiaceae*	*Rhodococcus rhodochrous*	
*Dietziaceae*	*Dietzia maris*	125 – ≥1,000
*Nocardiaceae*	*Rhodococcus erythropolis*, *R*. *ruber*	
*Microbacteriaceae*	*Clavibacter michiganensis*	125
*Nocardiaceae*	*Rhodococcus jostii*	

The least resistant strains (MIC = 125 mg/L) were members of the species *Clavibacter michiganensis* and *Rhodococcus jostii* and individual strains of the species *Dietzia maris*, *Rhodococcus erythropolis*, and *R*. *ruber*. The highest tolerance to IBP (MIC ≥ 1,000 mg/L) was observed for strains belonging to *Agromyces mediolanus*, *Brachybacterium faecium*, *B*. *paraconglomeratum*, *Corynebacterium variabile*, *Curtobacterium citreum*, *Dermacoccus nishinomiyaensis*, *Dietzia maris*, *Gordonia terrae*, *Rhodococcus cerastii*, *R*. *cercidiphylli*, *R*. *corynebacterioides*, *R*. *opacus*, *R*. *erythropolis*, *R*. *ruber*, *Micrococcus luteus*, *M*. *lylae*, *Nocardioides albus*, and *N*. *jensenii*. There is no direct correlation between the taxonomic affiliation of actinobacteria and IBP resistance. For further biodegradation experiments, 16 strains highly resistant to IBP (MIC ≥ 1,000 mg/L) were selected (Table 2).

**Table 2:** Percentage of IBP (100 mg/L) remaining during biodegradation experiment after 7 days of incubation of the actinobacterial strains in the RS medium.

Strain	% remaining
*Agromyces mediolanus* IEGM 860	96.3±0.78*
*Corynebacterium variabile* IEGM 824	97.2±1.12
*Dermacoccus nishinomiyaensis* IEGM 393	100.0±0.00
*Dietzia maris* IEGM 297	93.3±2.14*
*D*. *maris* IEGM 302	92.1±3.44*
*D*. *maris* IEGM 459	89.3±5.63*
*Gordonia terrae* IEGM 153	93.2±2.71*
*Nocardioides albus* IEGM 820	90.3±2.10**
*N*. *jensenii* IEGM 821	88.9±1.38**
*Rhodococcus cerastii* IEGM 1278	85.9±2.17**
*R*. *cercidiphylli* IEGM 1184	78.4±1.72***
*R*. *erythropolis* IEGM 501	81.4±0.97**
*R*. *erythropolis* IEGM 711	95.3±0.15**
*R*. *fascians* IEGM 1158	97.9±1.30
*R*. *ruber* IEGM 596	89.5±2.67**
*R*. *ruber* IEGM 477	95.6±2.82

The results are presented as mean ± standard deviation (n = 3). Mean values are significantly different from the control:

*p<0.05,

**p<0.01,

***p<0.001.

It is known that IBP is a non-antibiotic drug with bactericidal, fungicidal, and virucidal properties [62, 63]. Of medically significant Gram-positive bacteria, IBP inhibited the growth of *Staphylococcus aureus*, *S*. *epidermidis*, *S*. *saprophyticus*, *Bacillus cereus*, *B*. *subtilis*, and *M*. *luteus* at concentrations (MICs) within the 150–450 mg/L to 1,250 mg/L range and higher [61]. The bactericidal effect of IBP is associated with its amphipathic properties contributing to the insertion of the IBP molecule into cell membranes, leading to their destabilization and consequently to the disruption of its biological functions [64]. The IBP MICs detected indicate the pronounced resistance of natural actinobacterial strains.

According to our data, actinobacteria were unable to metabolize IBP as a sole carbon and energy source. The biodegradation of IBP was shown exclusively in cosubstrate cultivation [65]. To assess the ability of the selected actinobacterial strains to cometabolize IBP, bacterial cells were incubated in the mineral salt medium supplemented with 100 mg/L IBP and 0.1% glycerol (Table 2). The most promising biodegraders under IBP co-metabolism were *Rhodococcus cerastii* IEGM 1278, *R*. *cercidiphylli* IEGM 1184, and *R*. *erythropolis* IEGM 501; on day 7 of the experiment, the biodegradation was 14.1, 21.6, and 18.6%, respectively. In preliminary studies, NB was tested as a biodegradation medium; however, the removal of IBP was significantly less than in the mineral medium with an additional growth substrate. Interestingly, not all strains selected for their IBP resistance showed the ability to cometabolize it.

In the experiments comparing the biodegradation abilities of selected strains in the presence of various growth substrates, *R*. *cerastii* strain IEGM 1278 showed the highest IBP removal in the presence of 0.1% *n*-hexadecane (S1 Table). When glycerol, meat-peptone broth, and pentanol-1 were used as additional substrates, the IBP biodegradation was significantly (p<0.01) lower—from 14.1 to 27.4%. In the presence of other cosubstrates, the biodegradation of IBP was not observed.

IEGM 1278 (GenBank MG645192.1) is a strain of the plant-associated species *R*. *cerastii* [66]. IBP is a propionic acid derivative that belongs to the phenoxyalkanic acid family of growth regulators found in plant tissues [67]. Because IBP and plant growth regulators have similar chemical compositions, we can expect *R*. *cerastii* IEGM 1278 to degrade IBP. Furthermore, it is known that plant-associated actinobacteria (representatives of *Microbacterium* spp. in particular) are characterized by their high resistance and degradative activity to a polycyclic NSAID diclofenac [68, 69].

### Biodegradation of IBP by *R*. *cerastii* IEGM 1278

**Fig 1:**
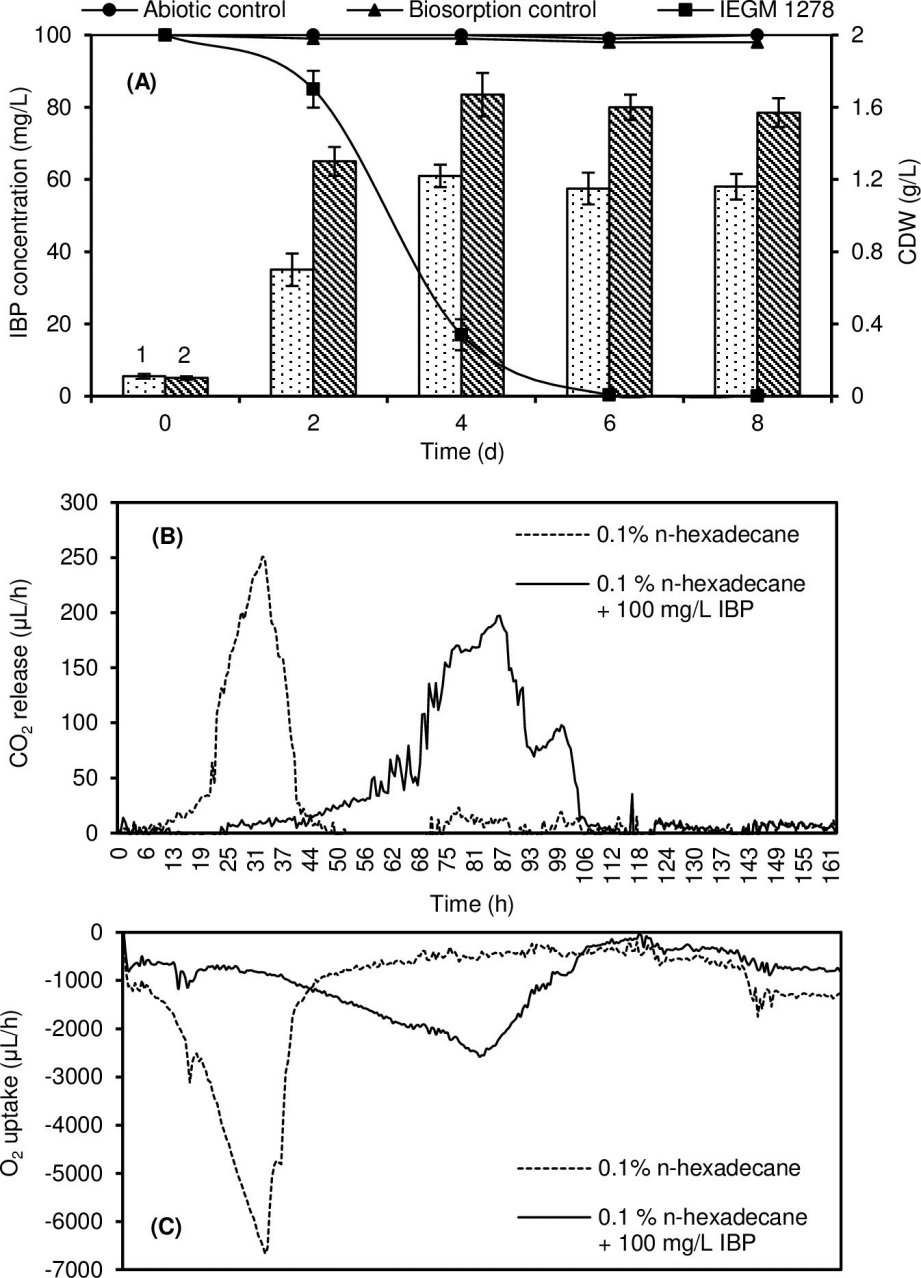
Biodegradation rate of IBP (A), carbon dioxide release (B) and oxygen uptake (C) by *R*. *cerastii* IEGM 1278. 1 –dry weight (CDW) of rhodococcal biomass in the presence of IBP and *n*-hexadecane; 2 –dry weight of rhodococcal biomass in the presence of *n*-hexadecane. Cells were pre-grown in NB for 3 days. Biodegradation experiments were conducted in the RS medium supplemented with 0.1% *n*-hexadecane. The graph shows mean values ± SD of three experiments done in triplicate.

The IBP biotransformation process was most effective using *R*. *cerastii* IEGM 1278 cells pre-grown in NB for 3 days and collected in the exponential growth phase (Fig 1A). Under such conditions, complete biotransformation of IBP was observed on day 6. The average rate of IBP bioconversion was 14.3 mg/day; the maximum values were reached on day 4 with an average rate of 21.65 mg/day. On the same day, the maximum of IBP degradation products was recorded (S1 Fig). The maximum specific rate of IBP biotransformation was 0.031 day^-1^. In the presence of IBP, the growth of rhodococci was significantly suppressed (p<0.05) (1.4 times) compared to the control variants.

When rhodococci were pre-incubated in NB for 1–2 days, the IBP bioconversion process was completed on day 8 of the experiment (S2 Fig). When using a four-day-old culture, the residual IBP in the medium was approximately 20% on day 8 of the experiment.

To assess the catalytic activity of bacteria in the process of bioconversion of complex organic substrates, it is advisable to use a respirometric analysis [70]. Respirometry can provide a reliable, repeatable, and technically sound assessment of microbial activity. The catalytic activity of *R*. *cerastii* IEGM 1278 to IBP was confirmed by data on oxygen consumption and carbon dioxide release. Fig 1В and 1C demonstrates that the maximum bacterial activity in the presence of only *n*-hexadecane occurs at 30–32 h of the experiment and then sharply decreases due to the substrate depletion in the medium. The maximum rates of O_2_ consumption and CO_2_ release reached -6667.3 and 250.6 μL/h, respectively. In the presence of IBP, a slowdown in the bacterial growth and activity was recorded in the first days of the experiment; the lag phase was about 70 h. The maximum metabolic activity (O_2_ uptake -2581.2 μL/h and CO_2_ release 197.2 μL/h) of IEGM 1278 cells in the presence of IBP was observed at 82–86 h of the experiment, which corresponded to the maximum rate of IBP biodegradation (see Fig 1A). Further decline in gas exchange in cells can be explained by the depletion of IBP in the medium and the accumulation of its metabolic products (see S1 Fig). The calculated average rates of oxygen uptake by *R*. *cerastii* IEGM 1278 with or without IBP were -1015.0 and -1230.0 μL/h, respectively. The total amounts of oxygen consumed in the presence of IBP and in the control were -218093 μL and -209770 μL, respectively.

The average values of carbon dioxide release rates in the presence of IBP were significantly (p<0.01) higher (1.5 times) than in the control: 36.3 and 23.8 μL/h, respectively. The total amounts of CO_2_ released by rhodococci were 4396.0 and 3857.4 μL with and without IBP, respectively. Thus, according to the carbon dioxide release, the metabolic activity of rhodococci was higher in the presence of IBP.

**Fig 2:**
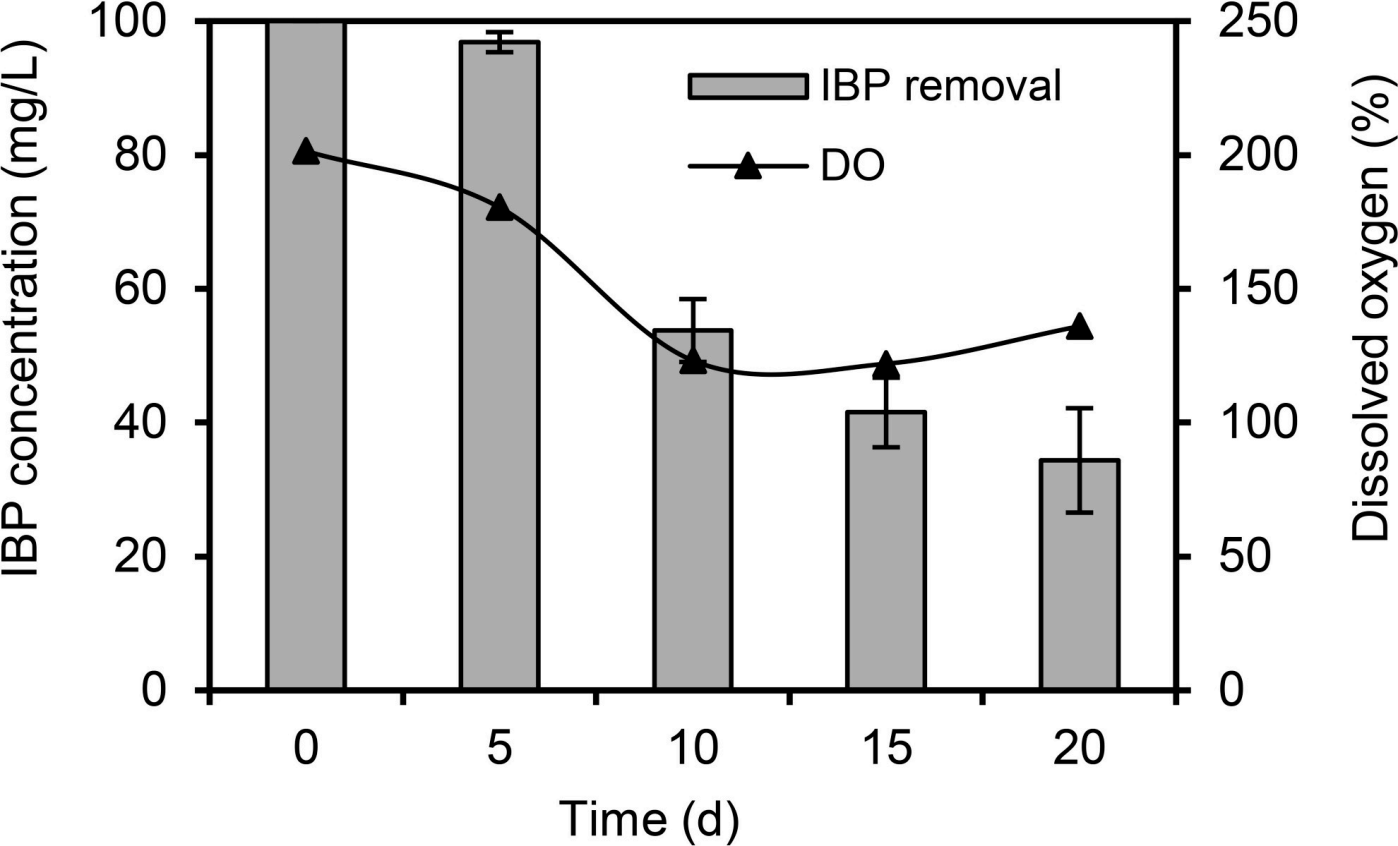
Biodegradation rate of IBP by *R*. *cerastii* IEGM 1278 in a laboratory bioreactor. Biodegradation experiments were conducted in the RS medium supplemented with 0.1% *n*-hexadecane. The graph shows mean values ± SD of three experiments.

During the process of IBP biotransformation in a laboratory bioreactor, a slowdown in IBP removal was observed. The residual IBP was still more than 30% on day 20 of the experiment (Fig 2). It should be noted that the level of IBP bioconversion correlated (-0.92) with a decrease in the concentration of dissolved oxygen. We supposed that oxygen uptake would decrease with the accumulation of IBP biotransformation products (S3 Fig). However, further studies are necessary to validate potential inhibitory effect of high concentrations of IBP metabolites on respiration of actively growing bacterial biomass.

Ascomycetes (*Aspergillus nidulans*, *Eurotium amstelodami*, *Bipolaris tetramera*), basidiomycetes (*Bjerkandera* sp., *Phanerochaete chrysosporium*, *Trametes versicolor*, *Ganoderma lucidum*, *Irpex lacteus*), and individual strains of Gram-negative bacteria (*Variovorax* sp., *Sphingomonas* sp.) and Gram-positive bacteria (*Bacillus thuringiensis*), microbial consortia (active sludge, soil and water consortia) as well as algae (*Navicula* sp., *Chlorella sorokiniana*, *C*. *pyrenoidosa*) were reported as IBP biodegraders [30, 65, 71–78]. In addition, single cases of monocultures of bacteria capable of effective IBP bioconversion have been reported. Thus, *Nocardia* sp. NRRL 5646 was capable of complete biodegradation of 1 g/L IBP within 5 days [79]. Biodegradation of IBP (5–25 mg/L) by *Bacillus thuringiensis* B1 under metabolic and cometabolic conditions (glucose, phenol, benzoate), including those in the presence of anthropogenic pollutants (2-nitrophenol, 4-nitrophenol) and heavy metals (Cu^2+^, Cd^2+^, Co^2+^, Cr^6+^, Hg^2+^) was shown in a series of studies [65, 80, 81]. Our findings on the duration of the IBP biotransformation process (6 days) by *R*. *cerastii* IEGM 1278 are comparable to the cases of bacterial decomposition of this pharmaceutical described previously in the literature. The most vivid example of effective IBP biodegradation is reported with *Micrococcus yunnanensis* KGP04 isolated from wastewater under optimized conditions: almost complete (90.87%) conversion of the substance (100 mg/L) following 12 h incubation [82].

**Fig 3:**
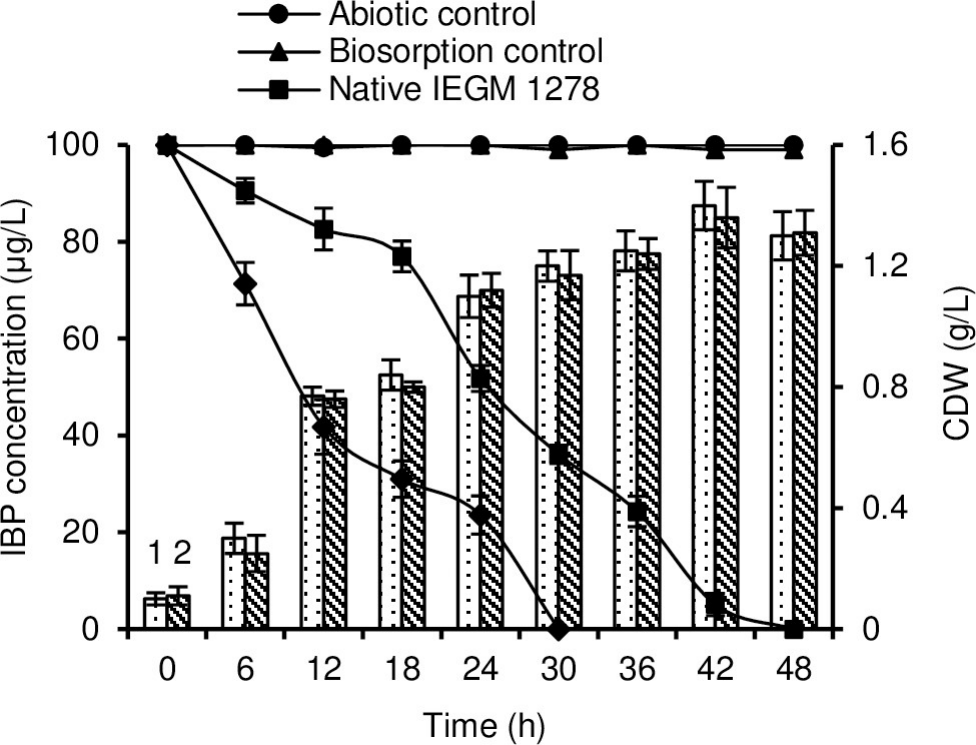
Biodegradation rate of IBP by native and pre-incubated *R*. *cerastii* IEGM 1278 cells. 1 –dry weight (CDW) of biomass in the presence of IBP and *n*-hexadecane; 2 –dry weight of biomass in the presence of *n*-hexadecane. Biodegradation experiments were conducted in the RS medium supplemented with 0.1% *n*-hexadecane. The graph gives mean values ± SD of three experiments done in triplicate.

In the environment, IBP is often detected in tens and hundreds of μg/L; therefore, the ability of the IBP degrading bacterial culture to oxidize the ecotoxicant in environmentally relevant concentrations is an important indicator of its biotechnological potential. We found that when added simultaneously with the inoculum (native cells), IBP was biotransformed by 100% within 48 h of the experiment (Fig 3). Rhodococci pre-incubated with *n*-hexadecane for 2 days performed the complete bioconversion of the above pharmaceutical in 30 h. During the experiment, an increase in biomass was noted in the control; the amount of biomass did not significantly (p>0.05) differ from that with IBP. Only a few studies on IBP bioconversion under environmentally relevant concentrations were previously described using monocultures or microbial consortia. Studies [83, 84] reported, for example, that *Patulibacter* sp. I11 was able to degrade 250 μg/L IBP by 50% in 300 h and 50 μg/L by 92% in 90 h. Nitrifying microbial consortia degraded 100 μg/L IBP in 24 h [85] or 72 h [86]. Using activated sludge, almost complete (94%) biodegradation of IBP was achieved on day 6 [86]. In a recent study [87], activated sludge degraded IBP (100 μg/L) after 36 h incubation.

### IBP biotransformation pathways

**Fig 4:**
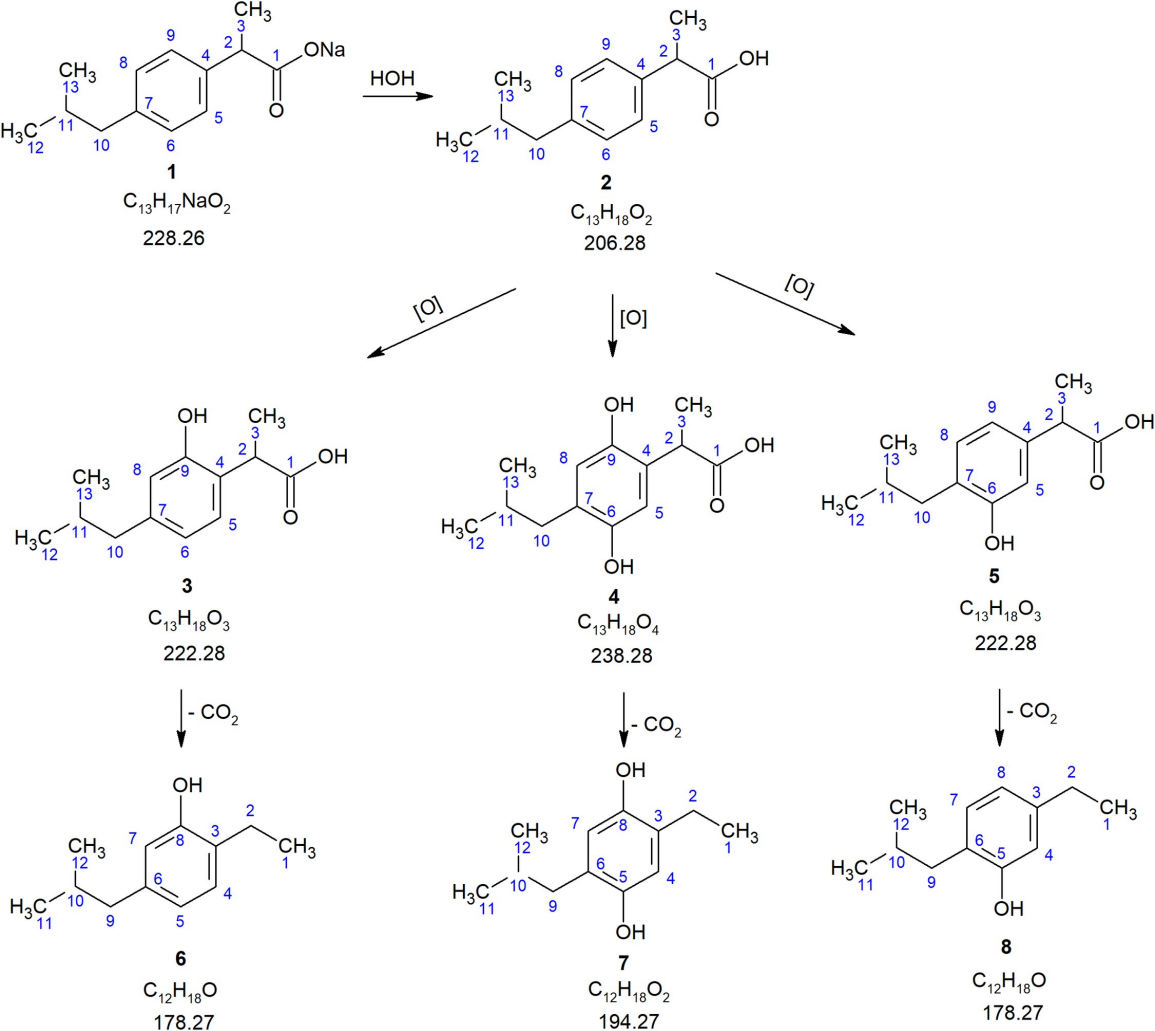
Proposed scheme of IBP biotransformation by *R*. *cerastii* IEGM 1278. **1** –ibuprofen sodium salt; **2** –ibuprofen; **3**–9-hydroxy ibuprofen; **4**–6,9-dihydroxy ibuprofen; **5**–6-hydroxy ibuprofen; **6** –decarboxylated derivative of 9-hydroxy ibuprofen; **7** –decarboxylated derivative of 6,9-dihydroxy ibuprofen; **8** –decarboxylated derivative of 6-hydroxy ibuprofen. The numbering of IBP atoms proposed by Preskar et al. is used [88].

Metabolites of microbial IBP transformation by the growing culture of *R*. *cerastii* IEGM 1278 were identified by LC-MS (Fig 4). The metabolites were detected in the selected ion monitoring mode (SIM) using the characteristic ions of the proposed IBP biotransformation products (S2 Table). In the first 2 days of the IEGM 1278 incubation in the presence of high (100 mg/L) IBP concentrations, its primary hydroxy metabolites were detected (compounds **3–5**) among the products of IBP biotransformation (compound **2**). When ions with *m/z* 222 (corresponding to the mass of the protonated molecule of a monohydroxy IBP derivative) were detected, two peaks with retention times of 2.67 min and 4.36 min were observed on the chromatogram (S4 Fig). These peaks belong to 9-hydroxy ibuprofen (compound **3**) and 6-hydroxy ibuprofen (compound **5**). Similar chemical structures of these compounds were confirmed by the mass spectra of the product ions obtained at different fragmentation energy values in the collision dissociation cell (S5 and S6 Figs). At the energy of the collision cell corresponding to 18 eV, an intense ion with *m/z* 149 was observed in both spectra. When the energy was increased to 35 eV, a more pronounced fragmentation of the precursor ion to product ions with *m/z* of 93, 121, and 149 occurred.

On day 4, decarboxylated derivatives of the above described hydroxy metabolites were detected in the incubation medium (compounds **6–8**). When ions with *m/z* 178 corresponding by mass to compounds **6** and **8** were detected, which are products of decarboxylation of IBP hydroxy derivatives, a peak with a retention time of 7.96 min was observed on the chromatogram (S7 Fig).

In the IR spectra of dry residues of IBP metabolites, the absorption band of phenolic hydroxyl was observed at 3410 cm^-1^. The presence of phenolic hydroxyl was also confirmed using iron (III) chloride. Hence, we propose that IBP transformation is accompanied by hydroxylation of the ecotoxicant molecule (compound **1**) to form 9-hydroxyibuprofen (compound **3**), 2,6-dihydroxy ibuprofen (compound **4**), and 6-hydroxy ibuprofen (compound **5**) and by their subsequent decarboxylation (compounds **6–8**). Identifying further transformations of hydroxylated and decarboxylated metabolites formed during IBP biotransformation requires additional research.

In contrast to IBP metabolization in the human body, the products of bacterial oxidation of the ecotoxicant are insufficiently studied. However, several studies reported and described individual products of the IBP biooxidation process as well as theoretical metabolic pathways of its biotransformation. For instance, Salgado et al. proposed a detailed scheme of actinobacterial metabolism of this pharmaceutical via two oxidation pathways with the formation of 22 products [84]. In general, IBP degradation was initiated by cleavage of the acid side chain to form catechols. Sharma et al. described a pathway of IBP degradation by *Micrococcus yunnanensis* KGP04 through demethylation, dealkylation, hydroxylation, and decarboxylation [82].

Most of the described metabolic pathways of bacterial IBP transformation affect the molecule’s aliphatic regions primarily. In contrast, the oxidation of the aromatic ring, which is necessary for degrading of the molecule’s structure, is usually observed at a later stage of the transformation process [16]. Of particular interest is the rhodococcal ability to oxidize the aromatic ring at the initial stage of IBP biotransformation. The introduction of substituents into the ring is essential for breaking its integrity and it is a critical step to further complete biodegradation of the compound [9, 10, 21].

It is known that the processes of bacterial oxidation of pharmaceutical pollutants involve enzymes localized mainly in the cytoplasm or associated with the cell membrane [16, 89, 90]. To determine the spatial location of enzymes that catalyze the biooxidation of IBP, its transformation was tested using various cell fractions. It was found that on day 4 of the experiment, the fractions of membrane-bound enzymes did not show IBP degradation activity, while the cytoplasmic fraction showed a moderate (up to 15%) ability to oxidize IBP compared to the growing culture (up to 90%). However, a peak corresponding to 9-hydroxyibuprofen was recorded by HPLC (Figs 4 and S8). Thus, the initial oxidation of the IBP molecule, accompanied by the incorporation of hydroxyl into the aromatic ring and the formation of primary monohydroxy derivatives (compounds **3** and **5**), is catalyzed by monooxygenases localized in the cytoplasm.

### Toxicity and characteristics of IBP biotransformation products

Though the toxicity of IBP itself is quite well studied using various test organisms, research on acute, phyto- and ecotoxicity of its bacterial degradation products is scarce and mainly limited to *in silico* studies. Using the QSARs software, Salgado et al. assumed that individual IBP oxidation products have pronounced toxicity to aquatic organisms and are classified as toxic in accordance with the Environment Agency Substances Information System [84].

According to our results, the 100 mg/L IBP had an inhibitory phytoeffect (37.2%) on the growth of plant roots (Table 3). However, the IBP biotransformation metabolites had more prominent (47.3%, p<0.001) phytotoxicity compared to IBP. Dilution (10^−1^–10^−3^) of IBP biotransformation products reduced their phytotoeffects (5.6–18.8%).

**Table 3:** Experimental phytotoxicity of IBP and its biotransformation products.

Variant	Root length, mm	Inhibitory phytoeffect, %	Test response
Control (water)	121.2±2.32	0	Norm
IBP 100 mg/L	76.1±3.11**	37.2	Inhibition
IBP biotransformation products	63.9±4.04**	47.3	Inhibition
IBP biotransformation products, 1/10	98.5±8.29*	18.8	Norm
IBP biotransformation products, 1/100	114.4±4.73	5.6	Norm
IBP biotransformation products, 1/1,000	111.5±1.87	8.0	Norm

The results are presented as mean ± standard deviation (n = 25). Mean values are significantly different from the control:

*p<0.01,

**p<0.001.

The ecotoxicity of IBP and its biotransformation products was predicted using ECOSAR (Table 4). ECOSAR analysis showed the final IBP metabolites (compounds **6–8**, *see* Fig 4) to be highly toxic compounds for aquatic organisms (fish, invertebrates, algae) because, according to the Global Harmonized System of Classification and Labelling of Chemicals, the values of acute and chronic toxicity of IBP transformation products to aquatic organisms are less than 1 mg/L [91].

**Table 4:** Toxicity of IBP (1–2) and its biotransformation products (3–7) calculated using ECOSAR.

Compound	Acute toxicity, mg/L			Hazard category	Chronic toxicity, mg/L			Hazard category
	Fish	Daphnia	Green algae		Fish	Daphnia	Green algae	
	LD_50_	LD_50_	ED_50_		(28 days)	(21 days)	(96 h)	
	(96 h)	(48 h)	(96 h)					
**1**	41.561	27.848	41.133	III	4.939	4.305	15.574	II
**2**	38.795	20.960	84.857	III	4.747	3.979	39.207	II
**3**	40.725	229.211	26.107	III	19.748	81.515	3.949	III
**4**	38.795	20.960	84.857	III	4.747	3.979	39.207	II
**5**	0.391	0.358	1.291	I	0.057	0.068	0.588	I
**6**	0.653	2.295	0.846	I	0.264	0.760	0.152	I
**7**	0.391	0.358	1.291	I	0.057	0.068	0.588	I

Compound numbers are presented according to Fig 4. The calculation of chronic toxicity is the geometric mean of the concentration leading to no visible effects (NOEC) and of the observed lowest effective concentration (LOEC). LD_50_ –the average lethal dose, ED_50_ –the average effective dose. I–highly toxic, II–toxic, III–dangerous for aquatic organisms, IV–non-toxic [91].

Using the EPI Suite software, the characteristics of IBP metabolites were predicted, which allow assessing their possible ecological fate. The main parameters used are the ability of the compounds to bioconcentrate and bioaccumulate in living organisms. In this case, bioconcentration refers to the process of entering of a chemical compound into aquatic organisms from the environment by adsorption through the respiratory tract and skin. Bioaccumulation is a broader concept that includes absorption of substances by organisms in any way (diet, dermal, respiratory) from any source (water, bottom sediments, food) [92]. It is shown that decarboxylated products (compounds **6–8**) of IBP biotransformation are characterized by high (log K_ow_>4) lipophilicity values and accordingly, by significant (4,999; 6,552 L/kg) coefficients of soil sorption and bioconcentration in living organisms (431, 208 L/kg) (Table 5). Moreover, the highest bioaccumulation rates were found for IBP (437 L/kg wet-wt) and decarboxylated compounds **6** and **8** (133 L/kg wet-wt).

**Table 5:** Soil sorption, bioconcentration and bioaccumulation of IBP and its transformation products calculated using EPI Suite.

Compound*	log K_ow_	Soil sorption, L/kg	Bioconcentration		Bioaccumulation	
			log BCF	BCF, L/kg wet-wt	log BAF	BAF, L/kg wet-wt
**1**	3.97	422.2	0.50	3.16	2.641	437
**2**	3.97	422.2	0.50	3.16	2.641	437
**3**	3.31	553.4	0.50	3.16	1.77	59.3
**4**	2.83	725.3	0.50	3.16	0.93	8.59
**5**	3.31	553.4	0.50	3.16	1.77	59.3
**6**	4.50	4999	2.63	431	2.12	133
**7**	4.02	6552	2.32	208	1.23	17
**8**	4.50	4999	2.63	431	2.12	133

Compound numbers are presented according to Fig 4. Bioconcentration and bioaccumulation values were predicted for fish.

Modeling of the biodegradability of IBP and its biotransformation products showed that the metabolites formed under both aerobic and anaerobic conditions are not readily biodegradable substrates (Table 6). The obtained data make one reconsider the risks of contamination of natural ecosystems with IBP since the products of its incomplete bacterial oxidation—poorly studied to date—can pose a significantly greater threat *in vivo* than IBP.

**Table 6:** Biodegradability of IBP and its biotransformation products calculated using EPI Suite.

Compound	Aerobic		Anaerobic	Criteria
	BioWin 5	BioWin 6	BioWin 7	
**1**−**2**	0.1976	0.1521	0.0334	Does not biodegrade fast
**3**	0.2060	0.1404	0.2096	
**4**	0.2144	0.1295	0.3857	
**5**	0.2060	0.1404	0.2096	
**6**	0.1098	0.1128	-0.0176	
**7**	0.1182	0.1037	0.1585	
**8**	0.1098	0.1128	-0.0176	

Compound numbers are presented according to Fig 4. A probability greater than or equal to 0.5 indicates that a compound biodegrades fast; less than 0.5 indicates that a compound does not biodegrade fast.

### Changes in morphometric characteristics of *Rhodococcus cerastii* IEGM 1278 cells exposed to IBP

Under co-metabolism with *n*-hexadecane, the response of rhodococci to IBP leads to cell aggregation with the formation of separate multicellular conglomerates of uncertain shape up to 1.5 mm in size (S9–S11 Figs). This appears to be an adaptive mechanism of rhodococci, allowing the bacterial population to adapt in conditions where single cells cannot decompose IBP and its metabolites. Such a defense mechanism can be a precursor of the biofilm formation process. The formation of cell aggregates was noted earlier in the degradation of a polycyclic NSAID diclofenac [21].

**Fig 5:**
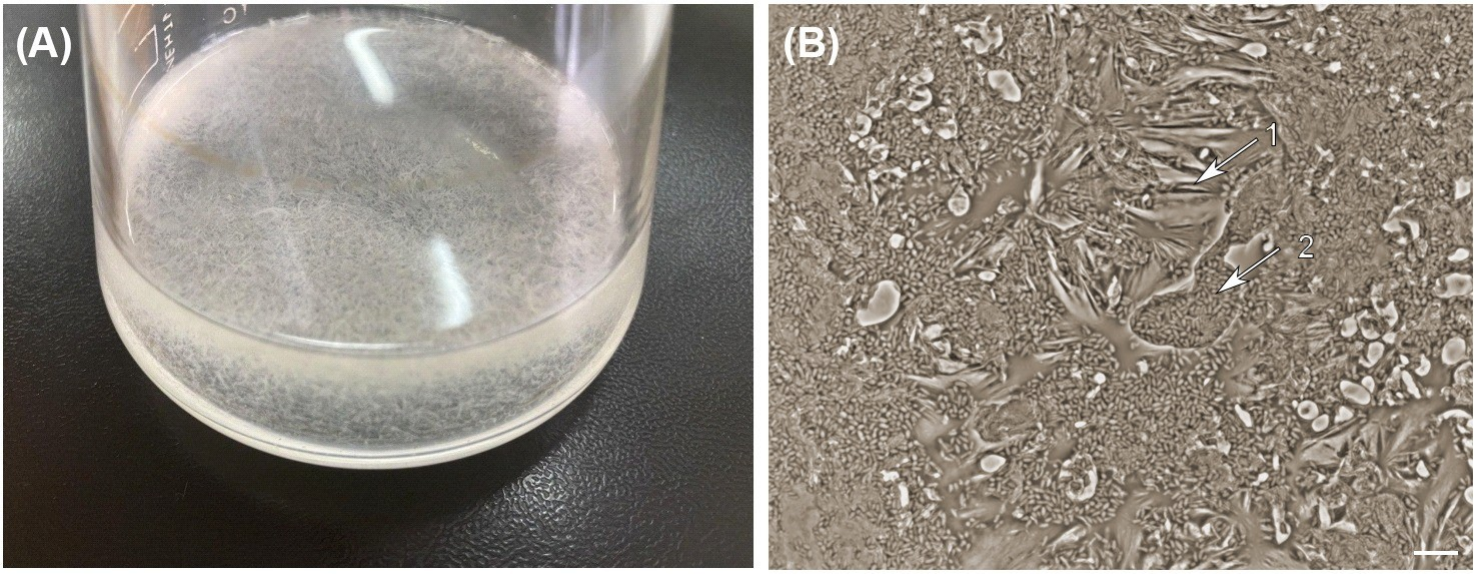
Cell aggregates of *R*. *cerastii* IEGM 1278. A–culture flask; B–phase-contrast image, x 1,000. 1 –cords; 2 –bacterial cells. Cells were grown for 3 days in the RS medium supplemented with 0.1% *n*-hexadecane and 100 mg/L IBP.

Aggregation of rhodococci was initiated on day 3 of the experiment and, apparently, was associated with the beginning of active IBP biotransformation (see Fig 1). In the first two days, bacterial turbidity was observed, and the optical density increased due to the active planktonic growth. The most characteristic feature of *R*. *cerastii* exposed to IBP was the formation of loose, needle-like, pale yellow aggregates on day 3 (Fig 5A). The absence of the bright orange pigmentation—typical of *R*. *cerastii* species—can probably be explained by the toxic effect of IBP, expressed by the inhibition of the carotenoid pigment biosynthesis [93]. Aggregates were clusters of cells surrounded by a polymeric cord-like extracellular matrix (Fig 5B). Similar structures were observed in the growth of Gram-positive bacteria *Staphylococcus aureus* [94]. It is known that the formation of cords in actinobacteria contributes to an increased degree of cell adhesion [95]. In the biotic control, planktonic culture growth was observed (S10 Fig). On exposure days 4–6, cell aggregation accelerated and bright orange bacterial clumps up to 15 mm in size were formed (S11 Fig). This was associated with IBP metabolites’ formation and consumption of *n*-hexadecane, and that bacteria entered the stationary growth phase. In the biotic control, as *n*-hexadecane was consumed on day 5 of incubation, the formation of brightly colored small dense aggregates was observed but with a significant number of single cells present.

**Fig 6:**
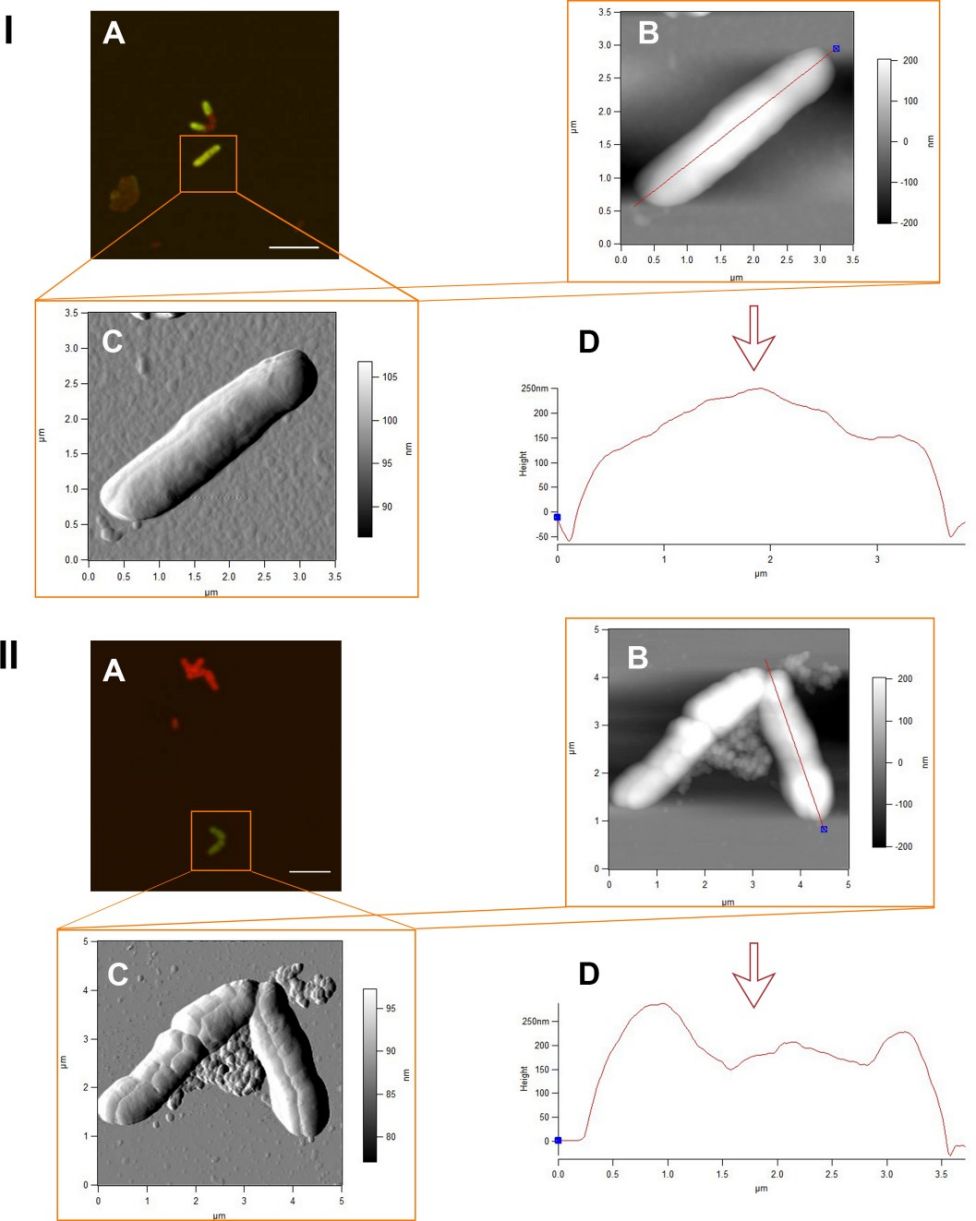
CLSM (A) and AFM (B, C) images and profiles (D) of *R*. *cerastii* IEGM 1278. Cells were grown for 24 h in the RS medium supplemented with 0.1% *n*-hexadecane (I) and 100 mg/L IBP and 0.1% *n*-hexadecane (II). The scale bars on the CLSM images correspond to 5 μm.

**Fig 7:**
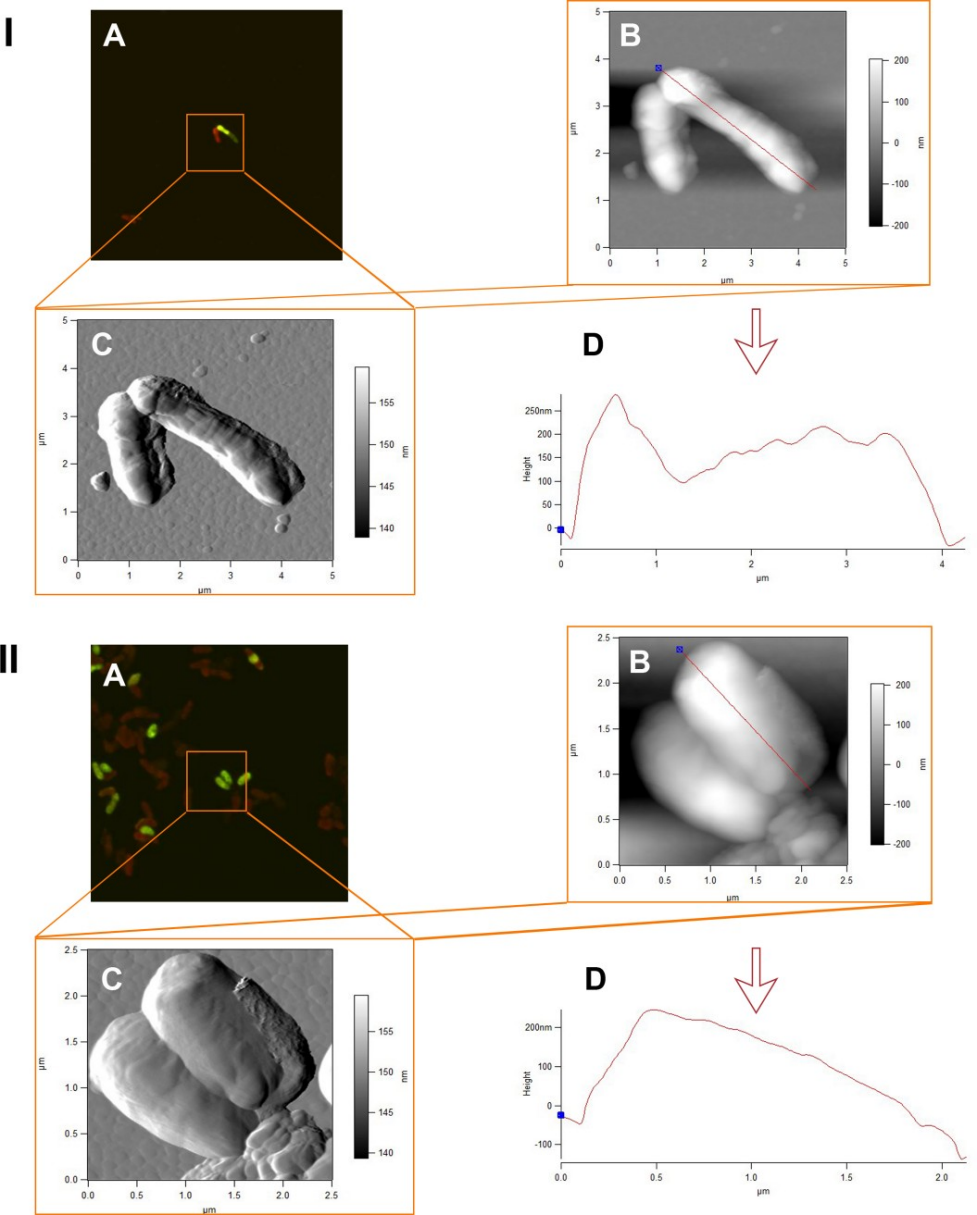
CLSM (A) and AFM (B, C) images and profiles (D) of *R*. *cerastii* IEGM 1278. Cells were grown for 4 days in the RS medium supplemented with 0.1% *n*-hexadecane (I) and 100 mg/L IBP and 0.1% *n*-hexadecane (II). The scale bars on the CLSM images correspond to 5 μm.

A system of combined atomic force and laser scanning microscopy allowed for generating accurate differentiated data on the size parameters and the relief features of living and dead rhodococcal cells exposed to IBP. The most interesting information was about the state of cells on day 4 of the experiment, which was associated with the active process of IBP bioconversion and accumulation of its transformation products in the culture medium. As shown in Table 7, in the first days of the experiment, there was a decrease in the cell length (p<0.05) and surface area (p<0.01) in the presence of IBP compared to control variants, but there was an increase (not significant) in the root-mean-square roughness of the cell surface (Table 7 and Fig 6). On the fourth day, the cells significantly changed their shapes due to shortening of the length (by 1.5 times) and increasing of the width (by 1.7 times) (Table 7 and Figs 7 and S12). At the same time, there was a significant (p<0.05) decrease in the cell surface-area-to-volume ratio (S/V) by 1.7 times, which is a defense mechanism for the presence of toxic IBP metabolites in the medium. Moreover, a decrease in the roughness of the cell surface exposed to contact with the ecostressor was recorded (Fig 7).

**Table 7:** Morphometric parameters of *R*. *cerastii* IEGM 1278 cells grown in the RS medium supplemented with IBP and *n*-hexadecane.

Variant	Length, μm	Width, μm	Volume, V, μm^3^	Area, S, μm^2^	S/V, μm^-1^	Roughness, nm
1 day						
0.1% *n*-hexadecane (control)	3.8±0.28	1.0±0.14	3.0±0.12	13.5±0.17	4.5±0.15	133.5±18.49
100 mg/L IBP + 0.1% *n*-hexadecane	3.2±0.21*	1.1±0.09	3.0±0.10	13.0±0.09**	4.3±0.11	169.6±25.54
4 days						
0.1% *n*-hexadecane (control)	3.5±0.42	0.6±0.21	1.0±0.14	7.2±0.15	7.2±0.17	136.2±27.33
100 mg/L IBP + 0.1% *n*-hexadecane	2.4±0.57*	1.0±0.13*	1.7±0.09**	7.5±0.10*	4.2±0.05*	121.6±36.17

Cells were cultured for 1 day and 4 days. The results are presented as mean ± standard deviation (n = 30). Mean values are significantly different from the control:

*p<0.05,

**p<0.01.

**Fig 8:**
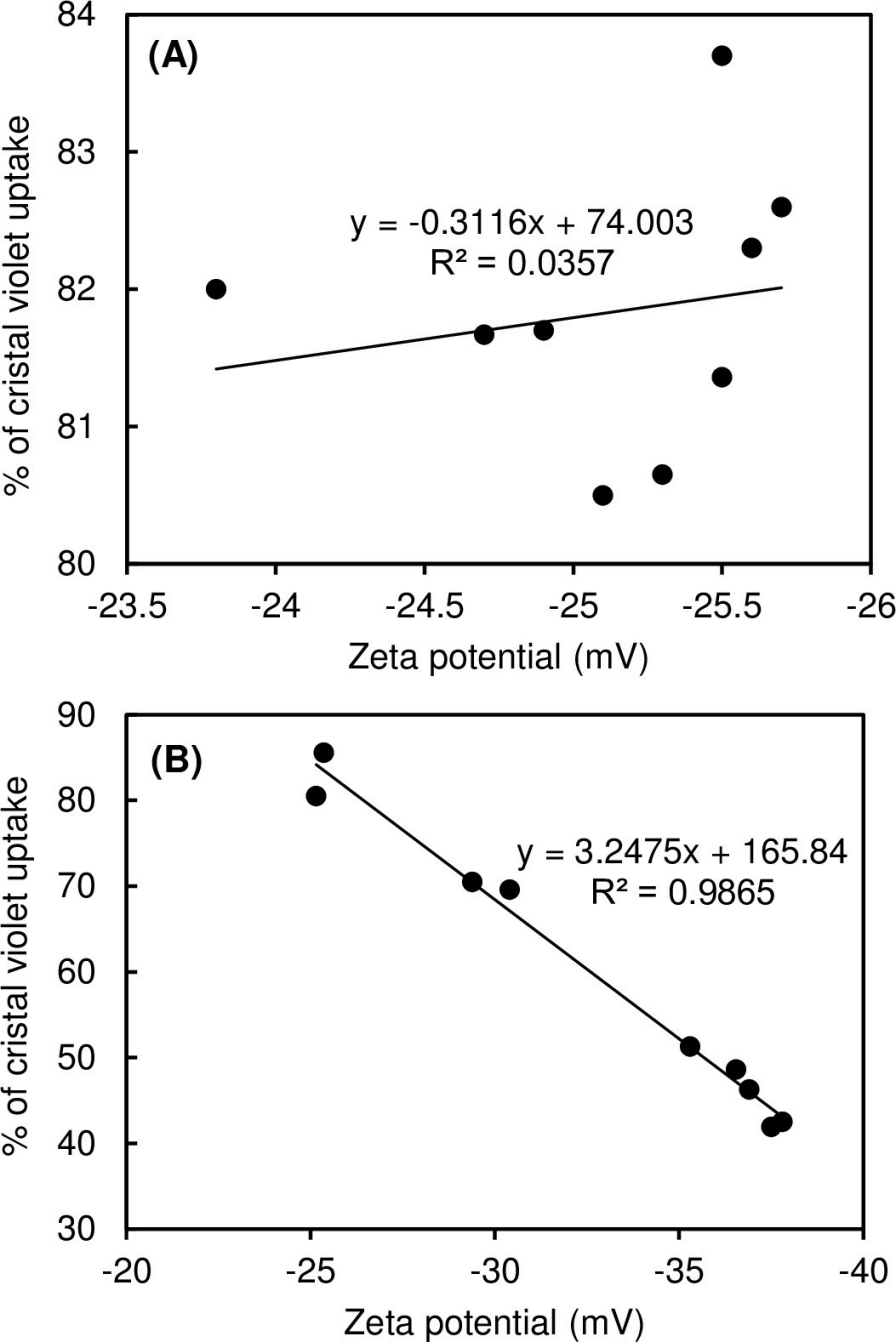
Correlation of membrane permeability with zeta potential of *R*. *cerastii* IEGM 1278. Cells were grown in the RS medium supplemented with 0.1% *n*-hexadecane (A) and 100 mg/L IBP and 0.1% *n*-hexadecane (B).

Another feature that expands our insights into the response of rhodococci to the presence of IBP is zeta potential of cell surfaces. The initial value of the electrokinetic potential of the rhodococcal cell surface was -25.1±1.4. On the fourth day of cultivation, zeta potential of rhodococci (-35.3±2.4) was significantly (p<0.001) by 10 units lower in comparison with the biotic control (-25.5±0.7). The shift to more negative zeta potential values may indicate a protective mechanism of cells for the presence of toxic products of IBP metabolism. We have previously shown that an increase in the negativity of the electrokinetic potential indicates the increased cellular aggregation of rhodococci in response to the presence of toxic and persistent diclofenac [21]. In this case, there was also a tendency to form cellular aggregates (S9–S11 Figs). In addition, a more negative cell charge may indicate the changed permeability of cell membranes in bacteria [96]. In the presence of IBP, as the negativity of zeta potential of cells increased, the permeability of their cell membranes decreased (Fig 8).

The obtained data on cellular aggregates formed, the shift of zeta potential to more negative values, and the decrease in membrane permeability are considered as mechanisms of *R*. *cerastii* adaptation and, consequently, an increase in their resistance to adverse IBP effects.

## Conclusion

Though IBP is one of the most frequently detected pharma pollutants in the environment, its metabolic pathways are not thoroughly studied yet, and the toxic effect of IBP on natural bacteria—potential biooxidants—is not virtually explored. A large-scale (100 strains) screening of actinobacteria from the Regional Specialised Collection of Alkanotrophic Microorganisms (IEGM, http://www.iegmcol.ru) resulted in the selection of *R*. *cerastii* IEGM 1278 with high (MIC ≥ 1,000 mg/L) resistance to IBP. This strain is capable of complete transformation of 100 μg/L and 100 mg/L IBP in the presence of *n*-hexadecane (0.1 vol. %) for 30 and 144 h, respectively. Cytoplasmic enzyme complexes are involved in the process of IBP oxidation. Influenced by IBP and its metabolites, transition of rhodococci from single- to multicellular lifeforms was observed, accompanied by a pronounced morphological anomaly of cells (changes in their shape, size, and cell surface roughness), a shift of zeta potential to more negative values and a decrease in the permeability of cell membranes. The initial stages of IBP bioconversion by *R*. *cerastii* IEGM 1278 cells resulting in hydroxylated and decarboxylated derivatives were described.

The documented high toxicities of IBP and products of its incomplete oxidation indicate that current environmental risks associated with environmental pollution by this pharmaceutical are underestimated. The results obtained suggest the need for more detailed study of pathways of IBP metabolization not only under natural conditions for ecological risk assessment of IBP but also under industrial conditions using active biocatalysts for optimizing of pharmaceutical wastewater treatment and neutralizing of pharmaceutical waste.

Because pharmaceuticals and their metabolites are increasingly detected in the environment in recent decades, one of the leading social problems is the development of scenarios to minimize their adverse impacts on natural biota and natural ecosystems’ sustainability. Hence, future research in this area should focus on overcoming these problems and taking adequate measures to prevent and reduce environmental risks from pharmaceutical pollution. Employing modern genomic and bioinformatic tools, these studies should primarily be aimed at an in-depth study of specific features of the “pharmaceutical pollutant–microorganism” interactions, which is necessary both for understanding of the protection mechanisms of native microbiota from the actual harmful effects of anthropogenic ecotoxicants and for developing of practical ways to neutralize and remove them from aquatic and terrestrial ecosystems.

## Supporting information

S1 Fig Dynamics of IBP (1) and its metabolites (2) during biodegradation by *R*. *cerastii* cells IEGM 1278. Biodegradation experiments were conducted in the RS medium supplemented with 0.1% *n*-hexadecane. (●) control of abiotic degradation, (▲) control of biosorption. The graph gives mean values ± SD of three experiments done in triplicate.
(PDF)

S2 Fig Biodegradation rate of IBP by R. cerastii IEGM 1278 (■). Cells were pre-grown in NB for 1 (A), 2 (B) or 4 (C) days. 1 –dry weight (CDW) of rhodococcal biomass in the presence of IBP and n-hexadecane; 2 –dry weight of rhodococcal biomass in the presence of n-hexadecane. (●) control of abiotic degradation, (▲) control of biosorption. Biodegradation experiments were conducted in the RS medium supplemented with 0.1% n-hexadecane. The graph gives mean values ± SD of three experiments done in triplicate.
(PDF)

S3 Fig The total area of HPLC peaks of IBP biotransformation products under laboratory bioreactor conditions. (●) the content of dissolved oxygen in the medium.
(PDF)

S4 Fig Chromatogram of the culture fluid of rhodococci (SIM mode; m/z 222). (PDF)

S5 Fig Mass spectra of the product ions of the protonated molecule (m/z 222) obtained by scanning the peaks with retention times of 2.67 min (a) and 4.36 min (b) (the energy of the collision cell is 18 eV).
(PDF)

S6 Fig Mass spectra of the product ions of the protonated molecule (m/z 222) obtained by scanning the peaks with retention times of 2.67 min (a) and 4.36 min (b) (the energy of the collision cell is 35 eV).
(PDF)

S7 Fig Chromatogram of the culture fluid of rhodococci (SIM mode; m/z 178). (PDF)

S8 Fig Chromatogram of IBP and 9-hydroxy IBP. The biodegradation was performed by the cytoplasmic cell fraction. The detection was carried out using an LC Prominence 20A chromatograph (Shimadzu, Japan) equipped with a reversed-phase column Phenomenex Jupiter® 5u C18 300 A, 250×4.60 mm, 5 μm (Phenomenex, USA) and a diode-matrix detector (SPD-M20A). Mobile phase–phosphate buffer solution (pH 5.0)–acetonitrile (40:60), eluent flow– 0.5 mL/min, column temperature– 40°C, sample volume– 20 μl, and detection wavelength– 254 nm.
(PDF)

S9 Fig CLSM images of *R*. *cerastii* IEGM 1278. Cells were grown for 3 days in the RS medium supplemented with 0.1% *n*-hexadecane (A) and 100 mg/L IBP and 0.1% *n*-hexadecane (B). Viable cells are stained with Syto9 (green fluorescence).
(PDF)

S10 Fig Cellular aggregation of R. cerastii IEGM 1278 in the presence of 100 mg/L IBP (A) and without it (B). Cells were grown for 3 days in the RS medium supplemented with 0.1% n-hexadecane.
(PDF)

S11 Fig Cellular aggregation of R. cerastii IEGM 1278 in the presence of 100 mg/L IBP (A) and without it (B). Cells were grown for 8 days in the RS medium supplemented with 0.1% n-hexadecane.
(PDF)

S12 Fig AFM images of *R*. *cerastii* IEGM 1278. Cells were grown for 4 days in the RS medium supplemented with 0.1% *n*-hexadecane (A) and 100 mg/L IBP 0.1% *n*-hexadecane and (B).
(PDF)

S1 Table Biotransformation of 100 mg/L IBP in the presence of additional carbon sources. (PDF)

S2 Table IBP and putative products of its biotransformation by *R*. *cerastii* IEGM 1278. (PDF)

S1 Dataset (PDF)
