# Supplementary material for: Response of Rhodococcus cerastii IEGM 1278 to toxic effects of ibuprofen
Source: PLoS One. 2021 Nov 18;16(11):e0260032. doi: 10.1371/journal.pone.0260032 (PMC8601567; doi:10.1371/journal.pone.0260032)
Supplement: S1 Fig — Biodegradation experiments were conducted in the RS medium supplemented with 0.1% n-hexadecane. (●) control of abiotic degradation, (▲) control of biosorption. The graph gives mean values ± SD of three experiments done in triplicate. (PDF) [file pone.0260032.s001.pdf]

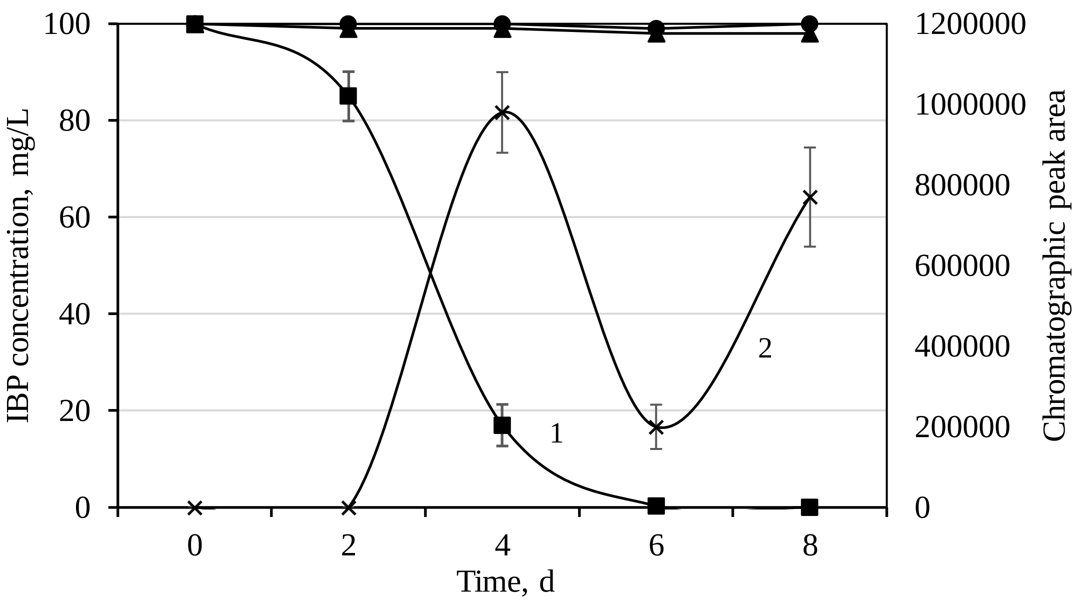

S1 Fig. Dynamics of IBP (1) and its metabolites (2) during biodegradation by *R. cerastii* cells IEGM 1278. Biodegradation experiments were conducted in the RS medium supplemented with 0.1% *n*-hexadecane. (●) control of abiotic degradation, (▲) control of biosorption. The graph gives mean values ± SD of three experiments done in triplicate.
