# Supplementary material for: Response of Rhodococcus cerastii IEGM 1278 to toxic effects of ibuprofen
Source: PLoS One. 2021 Nov 18;16(11):e0260032. doi: 10.1371/journal.pone.0260032 (PMC8601567; doi:10.1371/journal.pone.0260032)
Supplement: S2 Fig — Cells were pre-grown in NB for 1 (A), 2 (B) or 4 (C) days. 1 –dry weight (CDW) of rhodococcal biomass in the presence of IBP and n-hexadecane; 2 –dry weight of rhodococcal biomass in the presence of n-hexadecane. (●) control of abiotic degradation, (▲) control of biosorption. Biodegradation experiments were conducted in the RS medium supplemented with 0.1% n-hexadecane. The graph gives mean values ± SD of three experiments done in triplicate. (PDF) [file pone.0260032.s002.pdf]

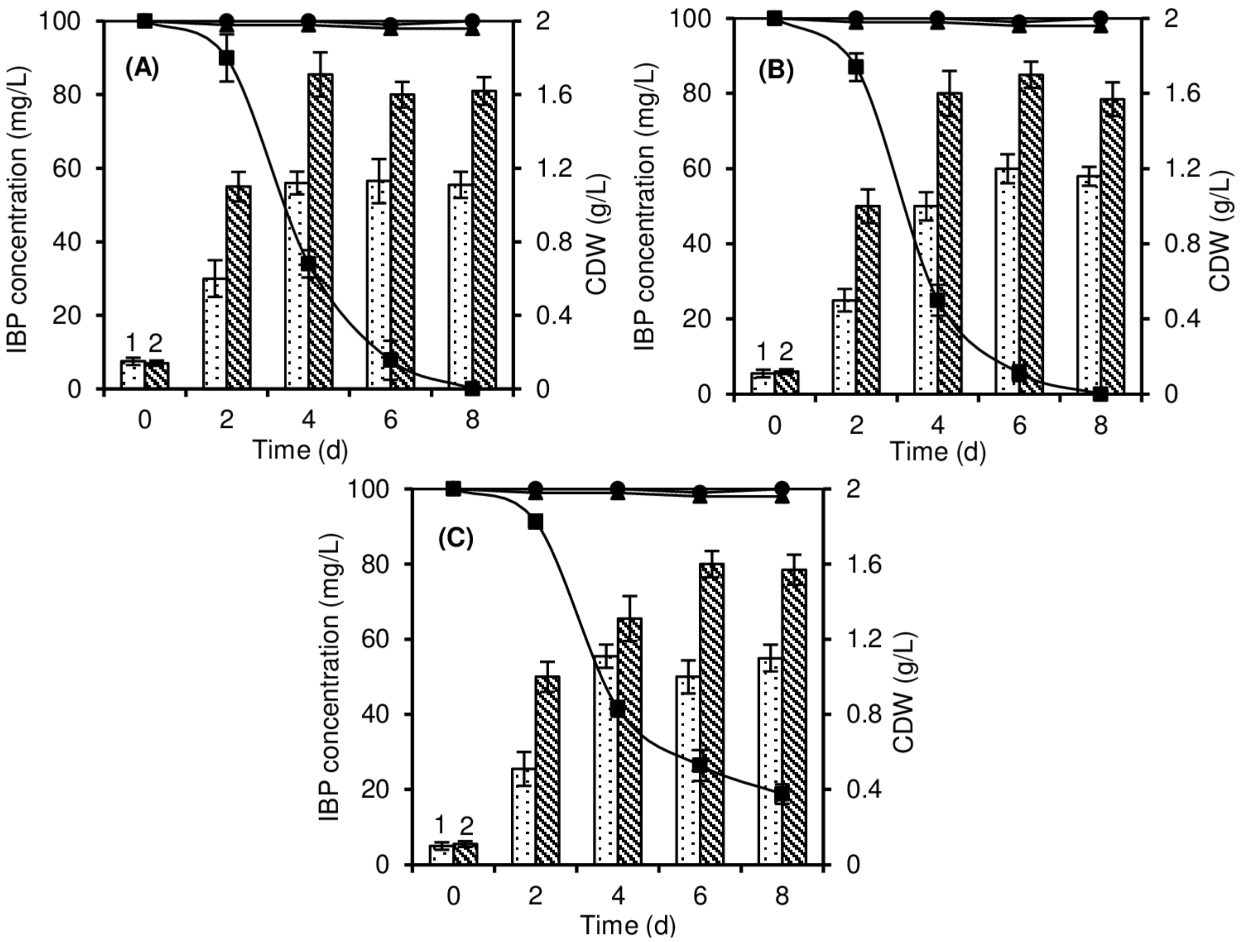

S2 Fig. Biodegradation rate of IBP by *R. cerastii* IEGM 1278 (■). Cells were pre-grown in NB for 1 (A), 2 (B) or 4 (C) days. 1 – dry weight (CDW) of rhodococcal biomass in the presence of IBP and *n*-hexadecane; 2 – dry weight of rhodococcal biomass in the presence of *n*-hexadecane. (●) control of abiotic degradation, (▲) control of biosorption. Biodegradation experiments were conducted in the RS medium supplemented with 0.1% *n*-hexadecane. The graph gives mean values ± SD of three experiments done in triplicate.
