# Supplementary material for: Response of Rhodococcus cerastii IEGM 1278 to toxic effects of ibuprofen
Source: PLoS One. 2021 Nov 18;16(11):e0260032. doi: 10.1371/journal.pone.0260032 (PMC8601567; doi:10.1371/journal.pone.0260032)
Supplement: S3 Fig — (●) the content of dissolved oxygen in the medium. (PDF) [file pone.0260032.s003.pdf]

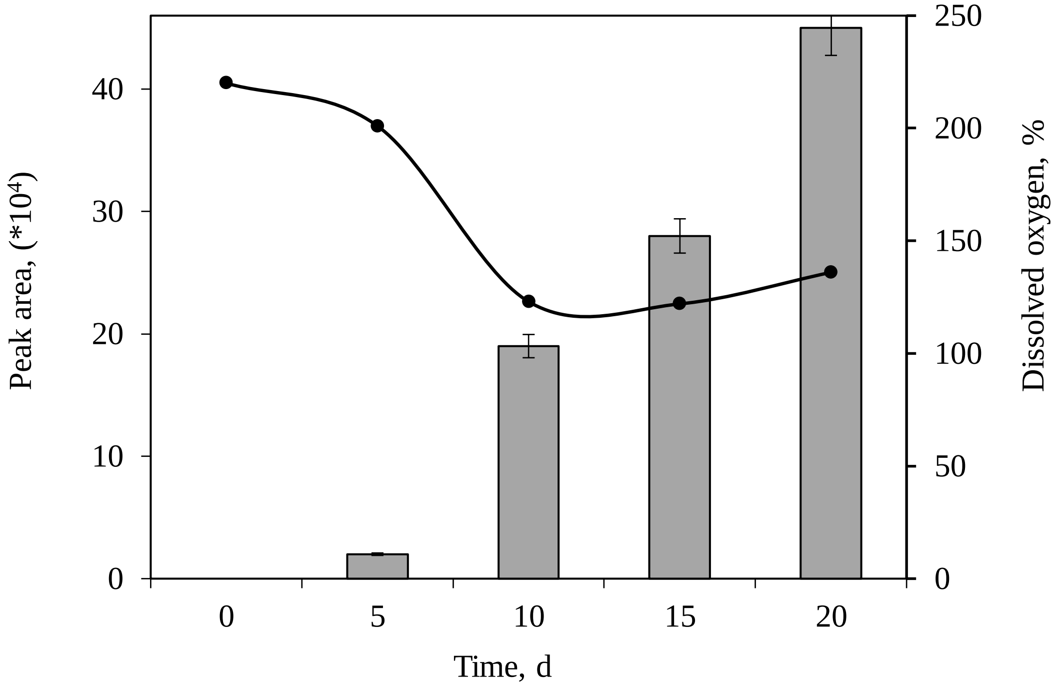

S3 Fig. The total area of HPLC peaks of IBP biotransformation products under laboratory bioreactor conditions. (●) the content of dissolved oxygen in the medium.
