# Supplementary figures and images for: Response of Rhodococcus cerastii IEGM 1278 to toxic effects of ibuprofen
Source: PLoS One. 2021 Nov 18;16(11):e0260032. doi: 10.1371/journal.pone.0260032 (PMC8601567; doi:10.1371/journal.pone.0260032)

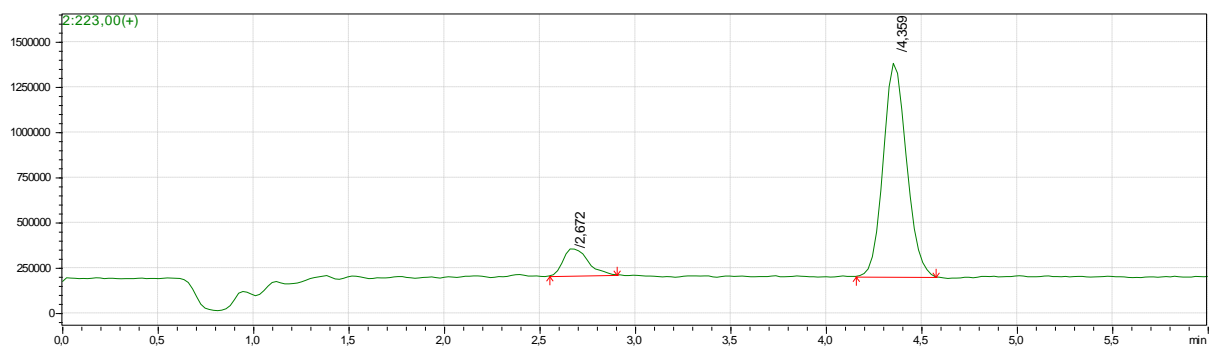

**S4 Fig. Chromatogram of the culture fluid of rhodococci (SIM mode; m/z 222).**

Supplement: S4 Fig — (PDF) [file pone.0260032.s004.pdf]

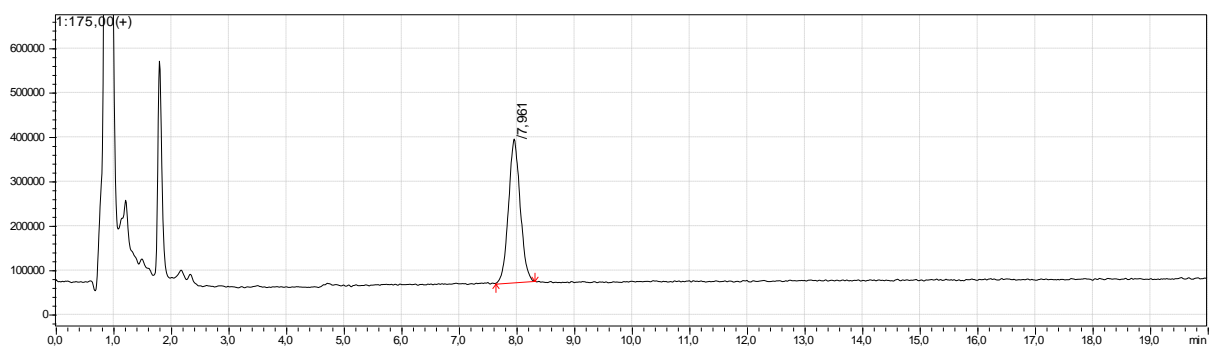

**S7 Fig. Chromatogram of the culture fluid of rhodococci (SIM mode; m/z 178).**

Supplement: S7 Fig — (PDF) [file pone.0260032.s007.pdf]
