# Supplementary material for: Response of Rhodococcus cerastii IEGM 1278 to toxic effects of ibuprofen
Source: PLoS One. 2021 Nov 18;16(11):e0260032. doi: 10.1371/journal.pone.0260032 (PMC8601567; doi:10.1371/journal.pone.0260032)
Supplement: S6 Fig — Mass spectra of the product ions of the protonated molecule (m/z 222) obtained by scanning the peaks with retention times of 2.67 min (a) and 4.36 min (b) (the energy of the collision cell is 35 eV). (PDF) [file pone.0260032.s006.pdf]

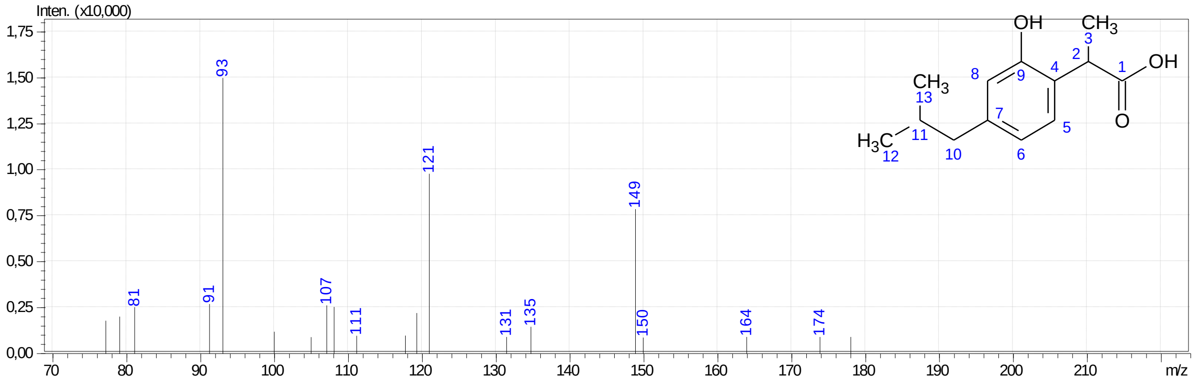

A

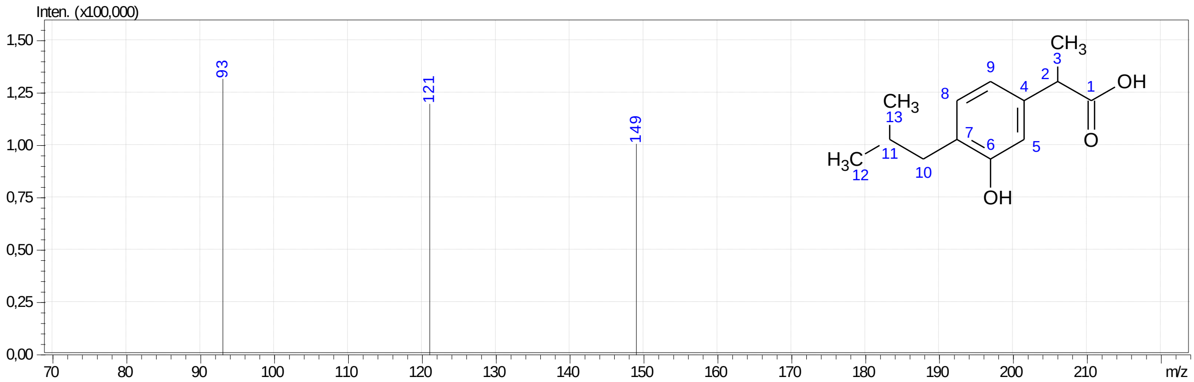

B

S6 Fig. Mass spectra of the product ions of the protonated molecule (m/z 222) obtained by scanning the peaks with retention times of 2.67 min (a) and 4.36 min (b) (the energy of the collision cell is 35 eV).
