# Supplementary material for: Response of Rhodococcus cerastii IEGM 1278 to toxic effects of ibuprofen
Source: PLoS One. 2021 Nov 18;16(11):e0260032. doi: 10.1371/journal.pone.0260032 (PMC8601567; doi:10.1371/journal.pone.0260032)
Supplement: S8 Fig — The biodegradation was performed by the cytoplasmic cell fraction. The detection was carried out using an LC Prominence 20A chromatograph (Shimadzu, Japan) equipped with a reversed-phase column Phenomenex Jupiter® 5u C18 300 A, 250×4.60 mm, 5 μm (Phenomenex, USA) and a diode-matrix detector (SPD-M20A). Mobile phase–phosphate buffer solution (pH 5.0)–acetonitrile (40:60), eluent flow– 0.5 mL/min, column temperature– 40°C, sample volume– 20 μl, and detection wavelength– 254 nm. (PDF) [file pone.0260032.s008.pdf]

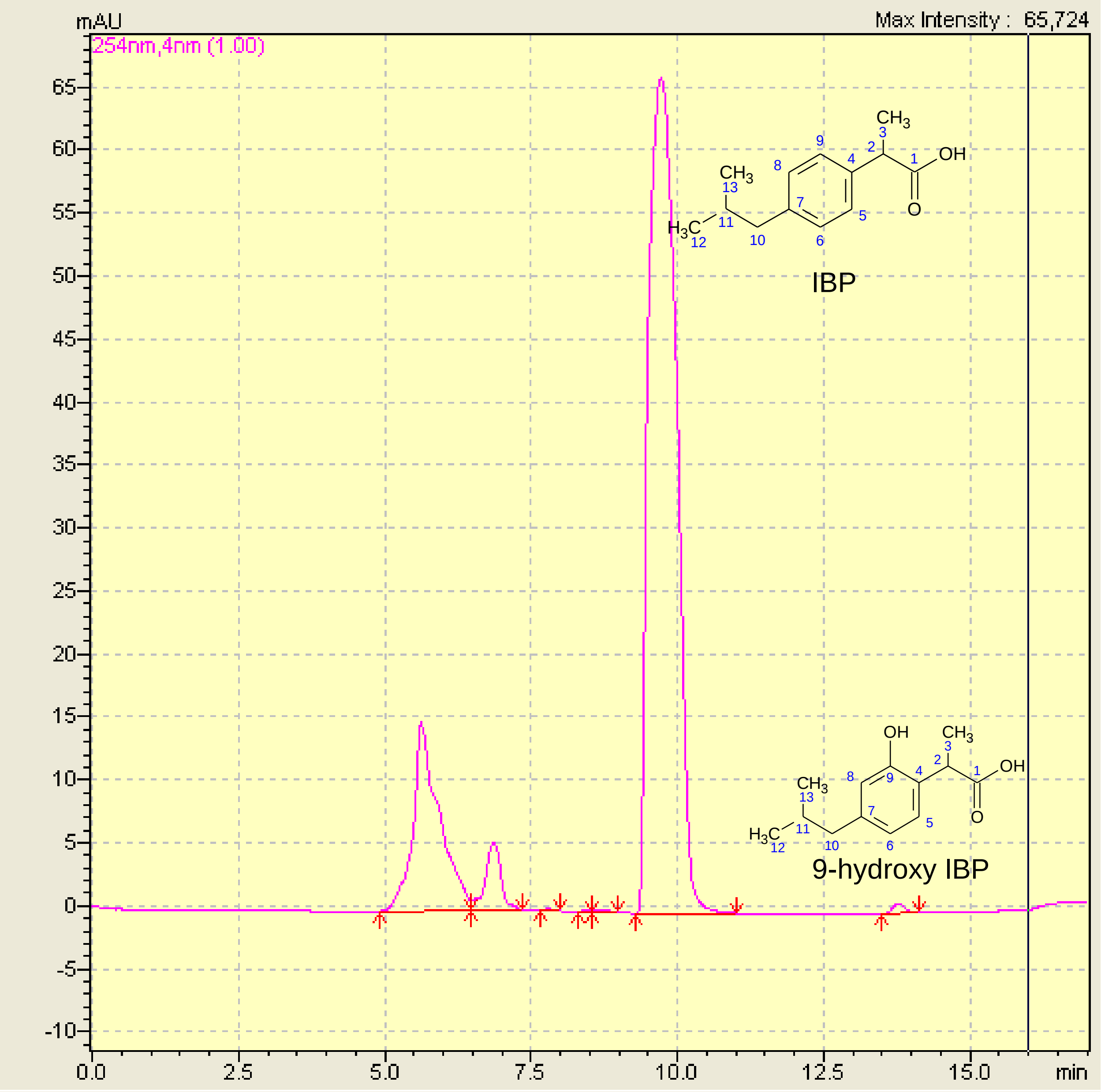


**S8 Fig. Chromatogram of IBP and 9-hydroxy IBP.** The biodegradation was performed by the cytoplasmic cell fraction. The detection was carried out using an LC Prominence 20A chromatograph (Shimadzu, Japan) equipped with a reversed-phase column Phenomenex Jupiter® 5u C18 300 A, 250×4.60 mm, 5 µm (Phenomenex, USA) and a diode-matrix detector (SPD-M20A). Mobile phase – phosphate buffer solution (pH 5.0)–acetonitrile (40:60), eluent flow – 0.5 mL/min, column temperature – 40°C, sample volume – 20 µl, and detection wavelength – 254 nm.
