# Supplementary material for: Response of Rhodococcus cerastii IEGM 1278 to toxic effects of ibuprofen
Source: PLoS One. 2021 Nov 18;16(11):e0260032. doi: 10.1371/journal.pone.0260032 (PMC8601567; doi:10.1371/journal.pone.0260032)
Supplement: S9 Fig — Cells were grown for 3 days in the RS medium supplemented with 0.1% n-hexadecane (A) and 100 mg/L IBP and 0.1% n-hexadecane (B). Viable cells are stained with Syto9 (green fluorescence). (PDF) [file pone.0260032.s009.pdf]

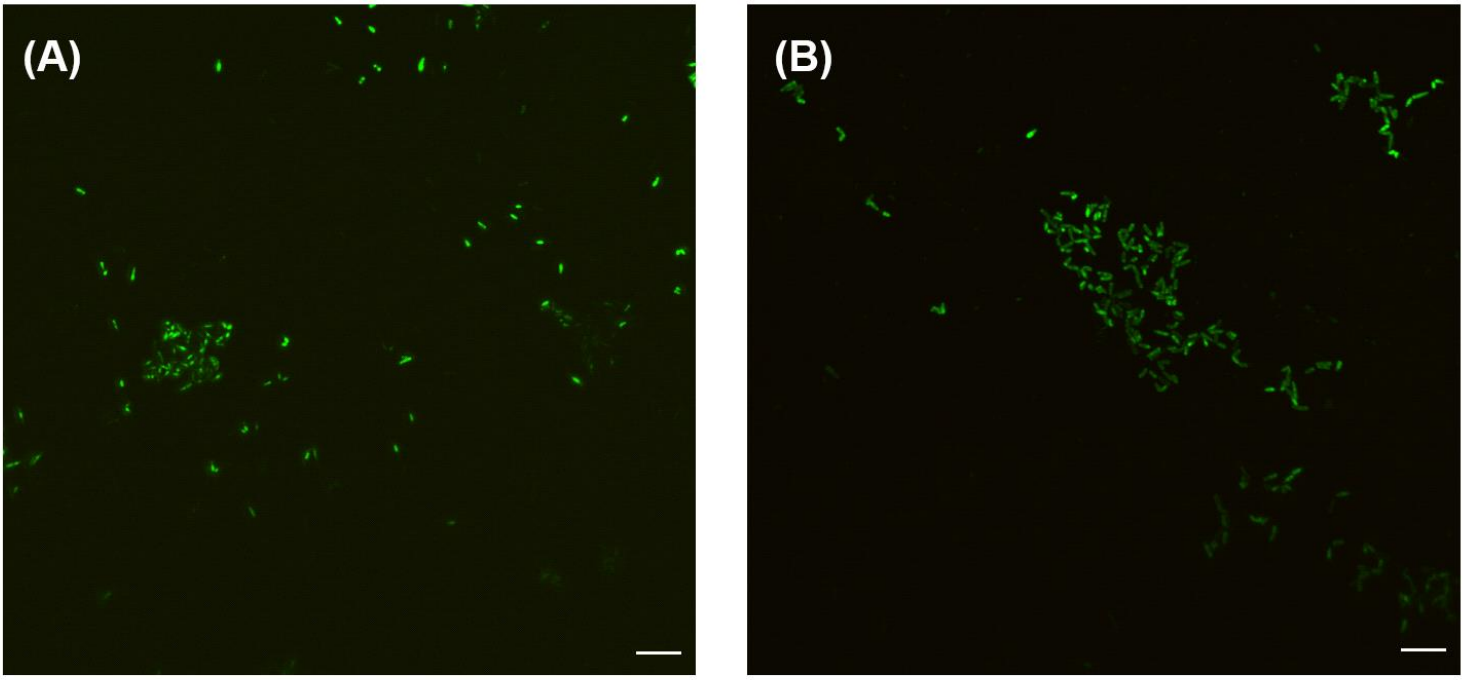

S9 Fig. CLSM images of *R. cerastii* IEGM 1278. Cells were grown for 3 days in the RS medium supplemented with 0.1% *n*-hexadecane (A) and 100 mg/L IBP and 0.1% *n*-hexadecane (B). Viable cells are stained with Syto9 (green fluorescence).
