# Supplementary material for: Response of Rhodococcus cerastii IEGM 1278 to toxic effects of ibuprofen
Source: PLoS One. 2021 Nov 18;16(11):e0260032. doi: 10.1371/journal.pone.0260032 (PMC8601567; doi:10.1371/journal.pone.0260032)
Supplement: S11 Fig — Cellular aggregation of R. cerastii IEGM 1278 in the presence of 100 mg/L IBP (A) and without it (B). Cells were grown for 8 days in the RS medium supplemented with 0.1% n-hexadecane. (PDF) [file pone.0260032.s011.pdf]

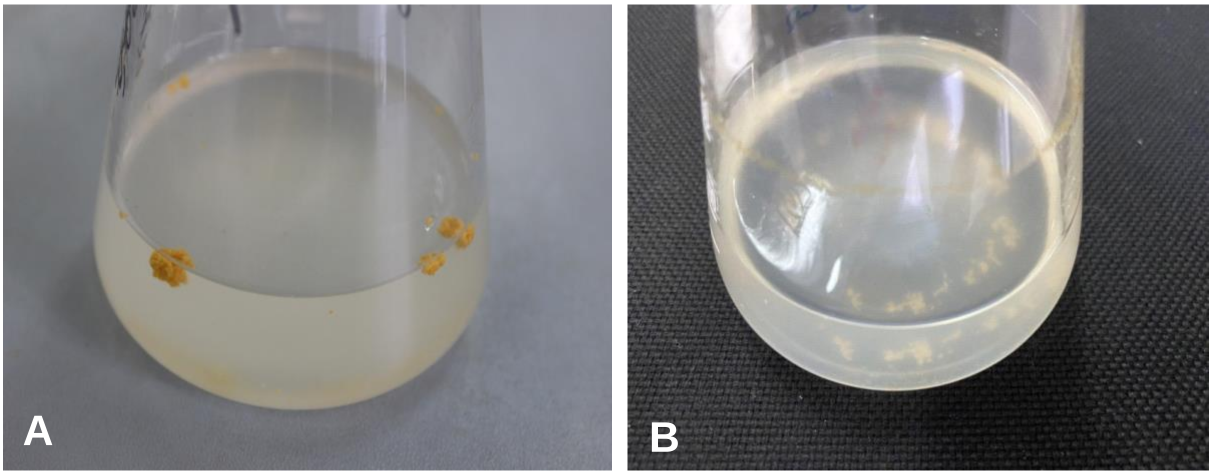

S11 Fig. Cellular aggregation of *R. cerastii* IEGM 1278 in the presence of 100 mg/L IBP (A) and without it (B). Cells were grown for 8 days in the RS medium supplemented with 0.1% *n*-hexadecane.
