# Supplementary material for: Response of Rhodococcus cerastii IEGM 1278 to toxic effects of ibuprofen
Source: PLoS One. 2021 Nov 18;16(11):e0260032. doi: 10.1371/journal.pone.0260032 (PMC8601567; doi:10.1371/journal.pone.0260032)
Supplement: S1 Table — (PDF) [file pone.0260032.s013.pdf]

S1 Table. Biotransformation of 100 mg/L IBP in the presence of additional carbon sources.

Strain	IBP decomposition, %									
	Glucose	Glycerol	Pentanol	Hexanol	Acetate	*n*-hexadecane	NB	Phenylacetic acid	Oleanolic acid	Humic acids
R. erythropolis IEGM 501	16.3±4.57	18.6±1.05	0	0	0	46.6±3.16	10.7±3.11	0	0	0
R. cercidiphyllii IEGM 1184	31.9±1.22	21.6±1.18	0	0	0	84.6±2.29	12.3±0.85	0	0	0
R. cerastii IEGM 1278	0	14.1±3.25	22.7±6.31	0	0	95.4±5.49	27.4±2.68	0	0	0

The results after 7 days of the experiment are presented.
