# Supplementary material for: Response of Rhodococcus cerastii IEGM 1278 to toxic effects of ibuprofen
Source: PLoS One. 2021 Nov 18;16(11):e0260032. doi: 10.1371/journal.pone.0260032 (PMC8601567; doi:10.1371/journal.pone.0260032)
Supplement: S2 Table — (PDF) [file pone.0260032.s014.pdf]

S2 Table. IBP and putative products of its biotransformation by *R. cerastii* IEGM 1278.

No.	Formula	Compound
**1**	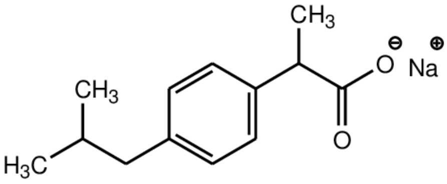	Ibuprofen Sodium Salt<br>$C_{13}H_{17}NaO_2$<br>MM 228.26
**2**	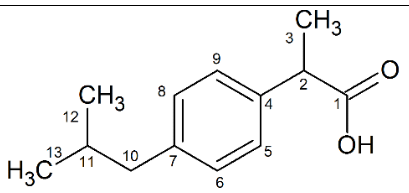	Ibuprofen<br>$C_{13}H_{18}O_2$<br>MM 206.28
**3**	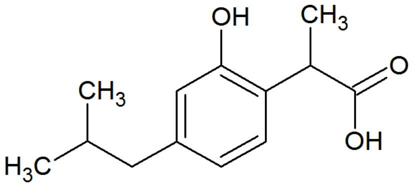	9-Hydroxyibuprofen<br>$C_{13}H_{18}O_3$<br>MM 222.28
**4**	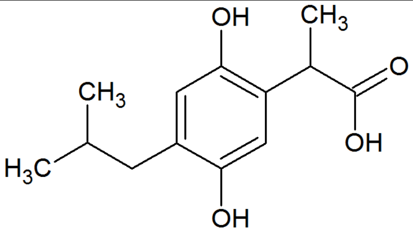	6,9-Dihydroxyibuprofen<br>$C_{13}H_{18}O_4$<br>MM 238.28
**5**	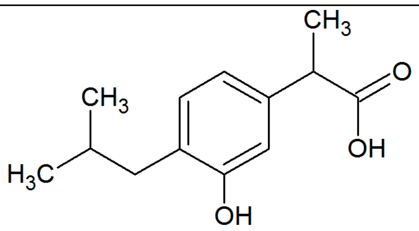	6-Hydroxyibuprofen<br>$C_{13}H_{18}O_3$<br>MM 222.28
**6**	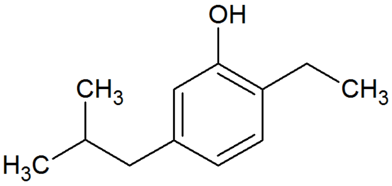	Decarboxylated derivative of 9-hydroxybuprofen<br>$C_{12}H_{18}O$<br>MM 178.27
**7**	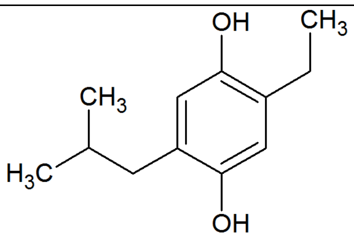	Decarboxylated derivative of 6,9-dihydroxyibuprofen<br>$C_{12}H_{18}O_2$<br>MM 194.27
**8**	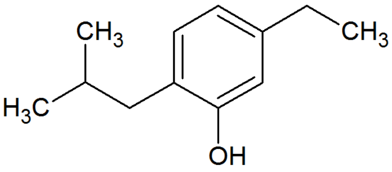	Decarboxylated derivative of 9-hydroxybuprofen<br>$C_{12}H_{18}O$<br>MM 178.27
