# Supplementary material for: Response of Rhodococcus cerastii IEGM 1278 to toxic effects of ibuprofen
Source: PLoS One. 2021 Nov 18;16(11):e0260032. doi: 10.1371/journal.pone.0260032 (PMC8601567; doi:10.1371/journal.pone.0260032)
Supplement: S1 Dataset — (PDF) [file pone.0260032.s015.pdf]

Minimal data set

Response of *Rhodococcus cerastii* IEGM 1278 to toxic effects of ibuprofen

Irina B. Ivshina*, Elena A. Tyumina, Grigory A. Bazhutin, Elena V. Vikhareva

Perm Federal Research Center of the Ural Branch of the Russian Academy of Sciences, Perm, Russia

*Corresponding author

E-mail: ivshina@iegm.ru (IBI)

Fig 1. Biodegradation rate of IBP, carbon dioxide release and oxygen uptake by *R. cerastii* IEGM 1278.

IBP degradation and cellular dry weight (CDW)

Time,d		0			2			4			6			8		
IBP concentration, mg/L	abiotic control	100	100	100	100	100	100	100	100	100	99.5	100	99.3	99.5	100	99.8
	biosorption control	100	100	100	98.9	100	99	98.5	98.2	99.1	98	98.6	98.1	97.4	98.6	98
	IEGM 1278 cells	100	100	100	85.7	87.3	85.2	13.7	17.8	19.9	0.3	0	0.7	0	0	0.1
CDW, g/L	*n*-hexadecane	0.11	0.1	0.12	0.66	0.82	0.65	1.09	1.33	1.24	1.15	1.22	1.09	1.13	1.25	1.1
	n-hexadecane + IBP	0.1	0.09	0.1	1.19	1.34	1.22	1.63	1.75	1.67	1.51	1.66	1.6	1.48	1.63	1.59

Respirometry

CO2 Rate						O2 Rate				
Time, h	IBP			Biotic control		IBP			Biotic control	
0.14	0	0	0	0	0	0	0	0	0	0
0.64	-2.069	5.763	1.301	-5.887	12.42	-3785.4	-1675.49	-821.936	-2582.99	-44.982
1.16	19.026	7.218	15.155	11.05	-1.279	674.775	1216.799	1338.394	1110.859	1139.078
1.68	7.68	12.198	7.601	6.488	0.106	556.322	1256.036	1186.014	1152.416	1053.335
2.19	3.84	3.661	-0.097	11.171	1.318	582.032	1114.188	1087.891	1279.211	1154.348
2.71	6.061	-7.683	-3.222	1.236	-5.613	461.763	1057.49	1076.792	1131.966	977.402
3.22	2.69	-3.532	0.551	8.166	-2.084	394.272	1005.501	1142.632	1244.819	1105.709
3.74	9.611	4.302	8.994	7.566	13.506	540.693	1149.48	1091.251	1189.336	1240.445
4.26	-0.707	1.342	-3.328	0.73	-2.816	196.396	857.898	930.189	893.175	1101.18
4.77	2.296	3.063	4.445	4.01	6.147	501.796	1059.383	1135.148	1164.241	1194.838
5.29	9.448	-1.099	-1.706	6.148	-3.112	410.116	885.694	1062.087	1119.853	1073.485
5.81	3.944	-5.384	-2.711	3.543	7.481	404.773	903.593	997.919	1131.925	1130.128
6.32	4.575	1.889	4.263	0.883	7.175	487.926	993.962	1111.653	1240.499	1288.989
6.84	-0.125	-6.824	-3.391	4.506	1.3	488.852	1057.619	1130.137	1308.566	1282.898
7.36	-1.313	1.01	3.94	1.982	11.068	497.933	1049.04	1114.664	1353.987	1358.368

7.87	6.57	2.403	2.686	6.082	10.274	435.003	1012.073	1031.041	1355.28	1368.572
8.39	2.949	1.325	-1.281	3.553	5.16	430.938	1028.601	1048.534	1411.214	1425.914
8.91	6.819	6.043	-0.634	7.944	2.411	455.639	1020.209	1117.038	1451.179	1410.042
9.42	4.409	2.697	4.774	11.248	6.15	451.733	1059.03	1114.359	1518.8	1415.079
9.94	2.288	0.528	-2.316	5.16	-0.655	476.977	1020.747	1104.163	1552.309	1544.593
10.46	0.641	-1.152	-0.658	4.062	10.772	502.801	1040.935	1142.427	1579.425	1615.221
10.97	5.25	0.608	4.402	5.286	11.768	463.78	1025.933	1120.021	1628.287	1672.427
11.49	6.015	4.468	2.483	11.739	13.978	502.638	975.402	1037.146	1650.622	1773.361
12.01	3.683	-6.447	-8.076	13.829	7.534	483.67	1028.964	1797.043	1769.797	1943.242
12.52	-2.156	-6.6	-1.788	8.786	12.154	565.716	2225.821	1921.754	1891.6	2037.426
13.04	-2.759	-2.07	-1.654	14.507	12.978	504.273	1098.842	1677.706	1997.871	2049.963
13.56	8.817	-8.294	3.166	20.811	17.856	676.892	1315.885	1306.125	2005.721	2284.968
14.07	7.352	-1.762	-3.941	15.44	17.863	621.721	1187.285	1966.025	2406.385	2055.593
14.59	-0.395	-10.959	-4.431	19.603	7.031	510.047	2012.675	2093.049	2320.041	2862.076
15.1	0.995	-0.238	-0.092	12.53	15.233	730.926	1502.223	1810.209	3205.944	3028.4
15.62	2.802	-5.64	-3.681	19.033	18.038	759.48	1887.938	1432.592	2472.93	2921.652
16.14	0.375	-12.17	-2.018	21.23	19.331	651.67	1559.463	1642.343	2747.289	2386.42
16.66	1.74	-12.315	-2.744	17.91	19.137	586.538	1501.137	1227.988	2431.435	2592.366
17.17	1.711	-8.258	0.963	18.053	23.829	671.822	1157.037	1292.32	2589.883	2769.052
17.69	1.441	-13.722	-5.311	26.258	24.882	609.831	1110.203	1356.412	2653.38	2586.398
18.21	1.588	-15.401	-2.354	27.493	27.439	592.524	1296.51	1259.884	2790.743	2746.28
18.72	6.812	-7.54	-3.152	29.684	33.508	703.506	1388.608	1007.291	2936.177	2814.784
19.24	6.784	-14.216	1.037	38.448	28.903	754.455	1100.005	999.21	3075.637	3062.512
19.75	-3.211	-9.06	-2.478	34.8	32.304	639.233	1134.499	1096.937	3069.711	3142.71
20.27	3.255	-25.437	-8.003	36.018	32.529	686.667	987.223	1142.553	3356.789	3320.936
20.79	4.951	-16.077	-3.436	33.962	34.368	760.383	1072.501	1204.725	3607.934	3324.942
21.31	1.152	-19.392	5.31	82.677	45.15	702.375	1017.07	1024.524	3573.891	3540.017
21.82	13.43	-14.303	-0.674	50.019	42.745	764.81	1230.021	1059.018	3799.93	3768.151
22.34	6.213	-20.664	-0.685	107.433	106.09	765.034	1156.038	983.451	4047.424	3913.889
22.85	6.661	-21.904	-5.624	121.788	112.904	777.744	1015.029	976.439	4143.634	4106.128
23.37	15.086	-7.221	8.446	138.873	123.69	799.55	1188.268	1116.652	4337.361	4228.995
23.89	5.691	3.131	-1.188	130.741	122.071	811.89	1020.39	1018.653	4360.944	4370.791
24.4	5.045	-12.547	-1.578	147.972	135.121	831.235	1077.355	1100.61	4580.401	4547.108
24.92	12.06	10.595	3.719	150.636	142.682	841.999	1125.996	998.19	4803.063	4638.175
25.44	10.008	3.497	4.971	166.819	159.449	856.072	1026.341	920.011	4900.263	4825.036
25.95	10.606	8.018	3.972	173.26	158.885	902.569	1110.002	931.771	5009.854	5053.56
26.47	12.617	4.647	2.068	178.479	168.801	944.828	1090.439	1240.452	5273.035	5050.03
26.99	7.197	10.301	0.342	182.521	176.745	875.534	1381.514	1034.933	5356.238	5194.321
27.5	10.228	3.198	10.74	193.714	191.319	969.67	1267.652	1118.856	5420.768	5442.625
28.02	19.875	9.292	5.994	199.42	201.566	1006.246	1128.827	1042.751	5581.952	5548.005
28.54	10.225	7.255	5.193	195.409	192.964	973.01	1127.937	1074.006	5717.836	5695.188
29.05	6.271	9.934	4.592	203.447	203.967	1018.497	1028.208	1108.188	5902.875	5776.333
29.57	8.332	10.737	2.615	203.587	218.862	1041.08	1145.027	1045.194	6014.919	5868.917
30.09	3.135	6.538	2.413	218.429	227.254	1024.553	1139.634	1104.175	6109.576	6083.978
30.61	13.304	6.818	4.603	229.001	235.704	1070.795	1060.663	1188.346	6336.153	6244.994
31.12	13.808	8.335	1.347	226.604	243.222	1084.565	1038.165	1184.115	6472.694	6313.044

31.64	11.564	8.464	6.968	224.927	252.527	1082.79	1103.423	1129.988	6629.313	6385.338
32.16	12.264	18.69	1.735	234.88	250.259	1130.166	1148.084	1085.691	6796.959	6537.682
32.67	14.595	7.524	4.099	240.152	261.108	1162.157	1237.114	1142.153	6994.69	6105.051
33.19	13.858	4.601	8.098	242.606	254.51	1174.692	1191.398	1108.565	7105.308	4425.315
33.71	12.276	10.823	7.703	244.051	219.205	1209.353	1118.708	1222.228	7288.926	2886.369
34.22	17.386	16.891	5.342	252.083	173.986	1228.503	1080.287	1228.835	7398.227	2254.884
34.74	9.37	6.88	2.633	255.175	127.141	1217.904	1158.879	1246.71	7551.702	1935.359
35.25	11	13.479	1.069	267.431	109.782	1200.373	1248.4	1185.012	7645.79	1912.94
35.77	16.145	14.56	10.016	267.46	93.841	1137.271	1148.422	1222.099	7738.696	1877.433
36.29	14.105	15.094	3.364	285.545	36.487	1222.801	1212.754	1318.194	5919.117	1740.971
36.8	13.613	7.539	9.789	289.321	29.56	1308.819	1257.946	1320.221	4055.314	1763.504
37.32	12.454	14.244	6.826	287.273	28.104	1340.842	1272.059	1335.625	3637.604	1708.807
37.84	15.385	9.612	5.953	256.528	24.409	1370.214	1357.121	1350.319	3047.15	1562.263
38.35	13.89	17.27	6.833	219.651	25.587	1396.281	1325.223	1444.515	2017.694	1509.662
38.87	15.174	17.203	7.81	175.11	19.501	1418.144	1331.658	1434.72	1734.03	1417.532
39.39	14.262	14.399	6.453	138.609	21.356	1449.153	1411.501	1564.517	1631.552	1380.567
39.9	23.537	12.76	6.976	120.68	18.601	1469.947	1379.138	1479.142	1524.633	1328.228
40.42	16.658	-6.125	3.243	40.742	13.97	1525.63	1474.405	1425.82	1498.606	1380.521
40.94	20.348	-4.019	11.532	36.6	18.071	1544.563	1533.69	1485.45	1423.631	1321.642
41.45	19.46	-0.583	4.731	22.713	10.676	1540.117	1511.2	1437.954	1294.998	1241.248
41.97	26.169	2.793	10.639	27.55	16.518	1580.783	1552.496	1510.287	1245.83	1165.124
42.49	21.759	-2.199	-0.227	26.078	5.735	1616.821	1513.702	1542.983	1095.285	1244.411
43	16.538	2.505	8.358	17.214	14.187	1680.737	1632.542	1612.294	1023.996	1213.715
43.52	22.503	12.724	13.324	16.444	15.41	1629.689	1545.175	1522.668	912.988	1047.438
44.04	24.692	9.822	9.301	8.228	6.052	1687.261	1615.633	1560.334	892.01	1166.52
44.55	24.014	13.797	10.756	7.772	9.157	1729.962	1611.636	1550.9	803.902	1140.901
45.07	23.027	14.668	11.274	7.434	4.198	1811.286	1648.112	1567.602	705.801	1150.059
45.59	26.725	16.881	12.868	12.435	9.542	1751.288	1508.211	1641.675	776.661	1052.589
46.1	27.706	9.627	11.056	4.722	4.076	1914.747	1697.932	1631.422	731.201	1152.121
46.62	29.664	12.436	11.921	5.89	7.418	1855.092	1624.611	1709.855	664.82	1065.347
47.14	26.529	19.15	21.235	0.342	4.572	1920.387	1626.545	1674.265	631.379	1107.475
47.65	25.338	20.762	17.786	5.081	-4.034	1946.223	1678.924	1717.53	667.181	1110.183
48.17	27.431	15.461	17.269	-1.941	1.809	1961.491	1734.59	1620.188	683.085	1014.693
48.69	35.345	20.755	19.005	8.163	10.973	1962.283	1768.056	1674.189	715.247	1068.172
49.2	34.682	18.044	15.831	-5.233	-1.638	2027.426	1833.548	1777.274	626.679	1028.616
49.72	26.308	23.495	15.85	-2.934	4.412	2072.549	1867.506	1862.775	654.251	989.064
50.24	34.629	22.228	17.063	-5.918	-5.655	2018.875	1881.984	1879.03	570.841	920.486
50.75	34.92	25.248	14.447	-0.207	0.791	2051.726	1938.353	1932.01	531.619	1032.535
51.27	35.446	27.025	24.963	-4.968	11.585	2058.93	1929.781	1854.093	546.826	1014.596
51.78	35.075	25.066	17.402	-6.907	-2.559	2063.332	1871.215	1856.956	583.058	980.91
52.3	32.738	21.646	16.979	-8.867	-0.331	2109.334	1903.832	2047.572	593.03	893.257
52.82	34.692	28.742	23.165	-4.947	-0.115	2094.979	1886.034	2028.161	530.012	856.601
53.33	39.418	31.165	24.109	-9.882	-0.06	2074.975	2023.119	2077.211	545.23	990.978
53.85	37.046	33.559	19.769	-8.527	-4.285	2039.477	1976.344	2109.678	574.918	945.953
54.37	39.331	26.896	19.676	-12.14	-1.615	2162.542	2088.468	2035.517	537.113	912.106
54.88	38.693	29.154	21.768	-5.927	-4.669	2171.509	2066.949	2073.851	537.604	910.682

55.4	38.315	32.354	21.693	-9.754	2.433	2176.185	2062.213	2151.985	531.395	840.412
55.91	39.277	29.38	19.323	-12.577	-10.409	2196.687	2151.02	2154.633	530.454	937.093
56.43	41.075	31.075	23.063	-5.734	-0.504	2209.778	2228.418	2120.314	504.9	914.796
56.95	41.219	32.268	19.921	-11.613	-13.725	2266.522	2234.294	2190.205	410.616	833.897
57.46	39.195	35.473	70.192	-9.333	-6.059	2341.183	2235.836	2093.639	494.371	951.856
57.98	85.97	36.191	30.19	-7.083	-1.812	2361.414	2271.393	2279.287	526.755	888.486
58.5	41.881	37.67	22.264	-10.627	-8.381	2302.376	2237.21	2318.997	431.504	730.105
59.01	38.94	36.554	25.452	-11.085	-9.481	2387.192	2390.355	2374.689	450.867	884.506
59.53	40.725	38.87	60.811	-13.75	-12.741	2433.934	2334.08	2230.876	534.756	823.484
60.05	45.859	43.171	24.934	-10.019	-3.337	2481.074	2278.309	2338.318	443.652	867.247
60.56	45.995	39.553	24.803	-9.363	-5.709	2479.775	2286.879	2409.267	389.611	885.894
61.08	46.91	36.078	25.268	-11.499	-8.624	2534.688	2518.61	2482.78	486.984	861.805
61.6	43.889	85.752	70.043	-15.488	-12.11	2606.378	2445.548	2370.92	561.87	651.906
62.11	48.641	42.203	76.118	-19.204	-11.405	2568.587	2435.418	2491.209	500.373	663.513
62.63	48.714	70.601	34.939	-15.5	-11.995	2570.301	2452.806	2534.083	439.957	712.835
63.15	92.941	87.204	30.632	-15.164	-10.212	2606.192	2475.469	2571.942	458.346	795.593
63.66	91.634	42.582	66.933	-17.605	-9.004	2620.563	2472.761	2532.95	513.963	797.231
64.18	51.648	50.7	56.424	-14.854	-3.859	2617.608	2558.486	2649.336	451.038	847.093
64.69	46.982	42.145	28.239	-22.738	-15.769	2701.002	2563.767	2602.266	410.007	806.147
65.21	52.937	45.357	73.556	-17.871	-11.769	2631.835	2512.938	2512.999	467.467	813.169
65.73	100.141	99.936	37.72	-18.844	-15.188	2760.768	2576.737	2670.569	560.993	779.535
66.25	55.593	47.997	56.922	-16.745	-12.285	2652.741	2530.312	2604.932	413.819	730.68
66.76	50.145	48.198	38.742	-18.759	-18.616	2609.162	2506.26	2569.616	349.655	627.03
67.28	59.184	51.082	41.859	-19.571	-5.96	2797.442	2515.274	2530.725	268.025	718.611
67.8	53.44	50.858	25.801	-21.658	1.217	2689.618	2543.502	2430.93	262.792	501.675
68.31	115.507	46.644	28.668	-38.661	-5.214	2788.939	2428.039	2461.202	127.626	661.169
68.83	118.602	120.322	83.532	-27.259	1.223	2880.649	2619.324	2463.906	369.115	756.738
69.35	130.093	107.583	86.952	2.204	-19.069	2971.656	2754.666	2664.64	356.881	803.997
69.86	129.706	63.984	36.221	8.619	-13.258	2833.683	2622.345	2472.031	299.564	707.547
70.38	156.085	132.635	117.386	-32.644	-2.796	2860.834	2540.205	2620.48	267.973	531.795
70.9	147.149	65.366	155.972	18.18	10.38	2934.917	2681.964	2866.441	397.727	744.98
71.41	131.883	110.959	100.696	-32.478	15.717	2851.494	2557.1	2609.709	106.459	767.459
71.93	125.657	121.274	161.392	9.802	-22.556	2922.104	2636.372	2763.104	384.155	560.742
72.44	119.05	72.592	142.765	9.128	15.236	2949.042	2526.99	2666.876	325.524	730.834
72.96	91.945	135.667	137.388	-17.497	11.462	3313.943	2521.423	2511.202	153.9	833.516
73.48	140.55	138.123	122.79	-13.341	13.481	3010.667	2742.629	2585.185	180.424	728.826
73.99	174.291	145.101	144.648	3.604	14.051	3055.209	2860.014	2976.285	21.11	582.158
74.51	151.307	154.127	148.759	9.786	10.875	3100.809	2923.839	2863.688	236.645	618.768
75.03	169.089	152.058	130.695	6.215	10.578	3204.988	2665.689	2822.692	162.814	604.959
75.54	165.153	158.792	174.063	12.565	21.805	3125.81	2649.206	2952.189	265.95	696.222
76.06	172.651	152.935	177.262	12.7	22.365	3262.304	2762.876	3040.08	401.359	805.826
76.58	179.513	161.671	169.277	13.68	21.606	3341.432	2812.729	3112.852	391.234	690.36
77.09	180.593	148.849	179.818	17.042	29.335	3325.965	2987.62	3063.934	383.825	588.969
77.61	171.64	148.37	171.792	12.172	17.1	3408.225	3028.355	3180.422	444.416	596.042
78.13	169.042	164.266	161.744	10.469	20.92	3418.867	3041.569	3147.146	392.171	576.069
78.64	170.761	157.739	171.274	7.749	18.357	3500.196	3100.725	3251.345	418.839	660.709

79.16	168.034	154.358	174.296	5.747	15.86	3522.197	2998.556	3300.152	405.727	649.26
79.68	162.248	159.213	174.046	9.958	14.247	3548.302	3102.528	3235.748	410.687	713.078
80.19	174.493	156.657	175.963	15.159	17.761	3636.718	3043.694	3335.35	429.226	711.383
80.71	169.69	160.197	175.008	5.605	9.616	3691.896	3205.494	3427.389	349.852	677.186
81.23	173.094	156.661	175.524	7.269	20.255	3610.781	3212.8	3377.562	219.103	589.25
81.74	176.648	153.367	177.08	7.62	13.031	3695.966	3242.914	3242.962	285.623	664.554
82.26	179.605	167.518	193.33	7.079	17.673	3747.463	3256.362	2669.727	275.948	623.498
82.78	172.335	164.631	206.05	0.539	9.452	3879.183	3244.414	2584.645	379.822	655.317
83.29	177.647	165.219	209.965	8.487	10.054	3860.196	3316.428	2419.706	310.23	594.803
83.81	185.289	167.402	207.377	4.906	13.22	3849.023	3235.063	2353.086	324.066	565.801
84.33	184.11	166.871	210.028	11.151	15.149	3859.756	3232.379	2279.08	251.103	590.25
84.84	182.815	178.859	205.089	3.7	11.358	3966.195	2778.66	2264.103	346.057	541.952
85.36	185.645	186.771	200.403	9.942	14.11	3929.668	2497.863	2302.897	261.08	595.958
85.87	189.036	200.217	201.464	6.344	11.718	3938.721	2357.753	2183.977	275.316	716.354
86.39	193.7	205.384	192.375	6.32	18.114	4052.287	2211.345	2072.286	350.928	530.576
86.91	181.422	202.493	183.053	6.404	14.306	4044.542	2230.631	1808.351	338.544	474.415
87.43	195.251	198.873	160.559	8.629	17.616	4121.159	2264.056	1509.236	329.296	479.282
87.94	204.913	203.596	136.387	6.686	17.674	4067.596	2192.244	1294.128	257.24	611.396
88.46	195.506	191.035	57.382	5.023	9.245	4138.885	2180.275	1049.32	309.39	553.561
88.97	197.684	191.926	59.08	5.739	-10.401	4110.423	2205.226	846.12	309.364	586.381
89.49	190.741	188.483	33.958	1.309	-15.684	4062.506	2069.995	625.645	262.108	550.747
90.01	184.761	190.807	40.128	1.263	-2.274	4201.196	2191.738	622.483	314.524	597.648
90.53	182.315	158.492	8.351	4.037	-4.985	4119.764	1806.324	439.864	211.125	524.493
91.04	201.7	170.761	14.581	-0.482	10.757	4047.42	1616.498	395.43	99.025	931.419
91.56	214.125	161.021	21.096	1.581	-3.498	4314.072	1474.164	409.434	335.68	502.628
92.07	210.02	66.72	10.135	11.567	3.478	4021.34	1053.821	259.294	196.293	502.432
92.59	205.971	47.736	-18.936	-1.053	-4.29	4022.038	644.406	109.771	218.662	258.134
93.11	210.757	38.189	-16.678	-1.609	6.832	4220.255	589.474	275.947	446.268	429.571
93.62	201.841	26.664	-7.181	-5.238	-0.676	3963.283	417.906	21.46	115.3	401.556
94.14	207.863	14.861	-14.088	-6.497	-21.509	3948.729	461.969	425.918	312.724	214.568
94.66	225.623	13.097	-2.444	-1.937	-6.384	3902.322	316.648	337.744	168.454	386.919
95.17	222.909	11.391	-8.972	5.068	-5.367	3995.879	261.071	42.838	163.855	463.866
95.69	231.963	3.587	-7.413	-6.794	0.74	4082.419	263.864	337.956	113.198	561.01
96.21	233.915	7.569	-5.488	14.646	7.041	3991.502	146.555	100.271	73.543	475.184
96.72	232.002	4.469	-4.43	11.693	-3.122	3879.583	285.29	161.695	388.307	440.883
97.24	244.713	3.995	-3.028	0.628	-1.315	3186.268	75.421	69.692	58.289	511.592
97.75	264.801	-5.738	-6.498	2.005	3.641	3134.807	179.67	117.137	245.948	525.107
98.27	266.284	-3.719	-6.965	10.446	3.36	3077.003	101.498	116.352	217.032	538.43
98.79	276.475	0.638	-3.084	6.442	0.844	3027.797	196.928	209.857	251.334	621.711
99.3	284.073	0.354	-0.823	13.735	6.578	3041.789	238.706	251.449	406.575	585.374
99.82	275.688	0.27	1.209	27.507	5.488	3000.562	309.635	236.639	408.365	590.001
100.34	283.112	4.626	6.105	22.175	15.883	3014.807	284.308	259.397	413.427	595.456
100.85	277.7	6.54	3.638	16.343	8.375	2949.411	193.892	200.852	331.645	562.101
101.37	263.809	-0.148	-2.479	8.041	4.205	2839.019	245.273	235.905	251.306	543.465
101.88	246.448	-3.036	-0.343	11.895	6.336	2560.812	247.601	280.043	308.851	530.371
102.4	223.197	3.628	2.926	9.635	9.085	2180.908	237.105	194.747	396.008	536.89

102.92	185.772	4.364	-4.784	12.127	3.172	1759.42	98.532	247.63	332.072	511.193
103.43	135.751	3.44	1.367	11.436	10.835	1468.796	153.738	300.164	379.739	518.096
103.95	112.004	5.615	2.741	12.297	14.127	1221.825	247.195	268.339	277.336	627.455
104.47	45.792	-2.663	-6.969	7.899	7.423	1093.525	233.927	201.261	305.869	618.039
104.99	40.226	4.012	0.447	-11.197	14.46	896.259	175.472	135.678	337.514	545.167
105.5	35.296	2.523	1.923	-9.374	6.241	824.473	180.636	168.244	304.884	392.576
106.02	23.777	-1.139	1.141	-9.314	2.607	805.516	143.934	238.904	314.798	565.94
106.53	20.483	-3.81	-0.022	1.5	8.424	656.78	259.706	169.394	274.626	574.014
107.05	20.65	3.282	-1.717	-2.246	6.286	688.963	153.944	294.651	263.78	552.47
107.57	21.112	-2.813	2.032	-1.19	4.451	645.049	201.914	235.155	191.351	516.801
108.08	22.493	-1.579	-1.181	-8.976	2.938	650.125	179.207	235.204	258.415	384.238
108.6	14.447	5.486	6.006	4.006	4.742	666.743	123.427	186.788	305.395	368.154
109.12	12.617	0.485	-0.812	-4.272	-3.086	604.638	172.411	204.604	333.759	447.243
109.63	9.02	1.27	1.387	-1.874	5.537	560.918	85.037	168.128	264.016	427.072
110.15	13.92	-0.921	2.369	-2.508	5.689	595.512	106.112	199.702	273.807	469.322
110.67	11.253	5.465	-7.489	1.146	8.684	568.197	-79.047	76.264	198.152	451.253
111.18	11.716	2.836	3.47	1.263	4.653	642.449	86.552	188.967	303.643	455.426
111.7	8.593	-0.852	-4.077	-5.021	2.645	617.556	150.108	54.792	281.004	389.888
112.22	5.778	-7.474	2.373	2.857	9.16	640.899	62.871	180.384	309.377	494.635
112.73	11.301	-0.192	1.681	0.837	11.219	644.087	-18.424	88.129	364.815	505.12
113.25	8.589	1.541	0.835	15.963	12.775	516.249	63.491	100.374	408.63	429.491
113.77	10.709	-16.943	-2.693	7.328	6.07	566.862	-201.688	196.089	518.699	440.694
114.28	0.022	-7.584	-5.202	2.422	10.295	478.951	127.932	46.903	276.959	179.364
114.8	9.803	0.938	1.025	3.933	5.648	468.603	-4.033	37.696	252.045	216.878
115.32	-2.351	-22.304	-17.01	-3.146	-2.071	502.227	3.282	126.394	384.488	395.02
115.83	2.435	-7.653	-3.12	-0.944	5.973	448.854	-145.216	225.134	308.581	448.686
116.35	20.277	36.617	48.664	19.494	36.32	417.671	-117.011	227.367	104.216	296.677
116.87	-20.551	-28.781	-45.313	-8.613	-44.464	226.528	-46.304	-42.437	485.425	421.295
117.38	-24.402	1.048	-3.408	8.431	21.206	263.919	116.328	-57.118	396.674	539.969
117.9	15.212	-16.301	-26.413	-32.262	-27.896	717.169	295.014	230.625	255.369	591.939
118.42	-31.357	-2.869	-14.099	6.575	-9.406	141.095	77.185	131.871	379.019	379.084
118.93	7.492	-8.174	-15.457	-8.583	-1.256	671.2	-144.297	-36.727	126.159	397.419
119.45	4.396	-9.653	-11.173	-9.474	2.393	437.037	47.472	37.497	113.262	584.088
119.96	-13.43	-9.656	-8.7	-26.3	-8.206	469.514	282.653	334.581	115.143	454.108
120.48	20.734	-2.59	-9.065	2.776	6.436	668.982	276.136	211.597	528.435	584.657
121	-1.179	-13.17	-11.969	-8.287	-4.315	546.76	208.96	191.768	363.055	548.471
121.51	27.825	-8.433	4.703	6.082	12.233	763.464	212.147	383.714	564.541	797.334
122.03	28.438	-2.432	1.245	25.054	0.589	748.529	260.48	324.798	521.085	738.871
122.55	23.197	6.606	10.292	2.535	16.784	867.319	427.081	501.286	555.109	729.371
123.06	19.75	4.759	4.222	7.318	6.505	824.873	297.915	338.685	425.709	621.639
123.58	12.682	-2.174	-0.133	2.25	9.539	772.156	300.419	361.173	415.16	697.002
124.09	21.453	0.08	4.722	4.474	16.045	773.451	368.115	324.201	535.836	649.678
124.61	16.517	-1.308	2.871	5.663	9.005	769.085	320.794	368.132	496.097	634.302
125.13	18.956	4.939	4.875	3.032	14.444	744.396	238.618	350.848	487.686	639.853
125.64	19.99	-0.027	6.24	7.631	15.139	719.682	281.98	424.797	415.919	667.792
126.16	12.819	0.029	4.459	0.953	6.117	760.387	347.908	280.15	445.272	651.799

126.68	16.337	-3.663	2.993	9.612	8.669	697.464	380.355	245.121	463.102	679.67
127.19	21.107	4.017	8.07	4.643	7.025	683.096	263.646	194.562	479.667	615
127.71	10.435	-0.619	6.164	-1.654	4.249	656.729	65.223	287.76	401.755	677.075
128.23	9.985	-2.12	1.727	4.851	8.842	685.848	97.209	304.002	421.189	738.315
128.74	12.16	-5.227	8.529	2.424	13.236	715.736	225.781	396.937	527.507	631.466
129.26	12.7	-0.74	-1.636	3.881	6.603	869.77	330.533	350.427	388.586	649.705
129.78	14.395	3.188	-1.415	-1.38	10.393	716.235	278.799	422.713	470.796	493.963
130.3	11.005	-1.207	-2.454	-1.147	7.162	733.973	340.439	470.486	378.714	551.702
130.81	17.046	0.19	2.023	-0.729	12.431	672.965	288.843	430.84	406.429	632.005
131.33	9.766	-1.397	0.434	-2.206	4.592	581.464	266.897	363.619	458.549	579.09
131.84	8.48	1.335	4.682	2.916	3.811	626.456	371.483	346.496	512.86	699.901
132.36	9.282	1.27	4.331	2.331	6.965	575.511	325.649	415.739	454.182	624.981
132.88	10.901	-5.306	0.125	-1.074	7.907	654.906	330.747	382.111	557.108	752.269
133.39	9.129	1.184	2.842	1.749	7.149	601.045	302.589	342.528	519.109	671.266
133.91	11.512	5.753	1.299	-2.961	5.599	565.254	313.47	449.835	496.184	675.8
134.43	10.014	-0.549	-1.177	1.325	2.206	584.985	380.466	383.739	541.147	796.952
134.95	4.147	4.392	-0.891	-3.002	6.771	639.86	395.217	601.338	581.253	861.49
135.46	9.699	-1.736	-1.297	5.504	4.935	531.626	354.599	413.089	517.47	716.783
135.98	7.633	-1.088	-0.759	2.978	3.921	491.146	493.744	472.834	535.05	779.168
136.5	4.43	0.097	3.805	0.435	7.019	565.306	497.025	508.534	728.053	754.18
137.01	6.912	1.893	2.42	1.683	4.434	580.538	634.364	543.978	647.342	605.654
137.53	8.849	-3.455	-2.344	3.415	-0.702	513.845	468.88	509.317	541.72	612.401
138.05	0.167	1.881	-1.059	6.142	8.797	536.83	646.383	492.139	537.448	915.257
138.56	11.358	-2.854	-3.318	5.427	7.653	484.039	676.656	582.167	617.37	820.783
139.08	-5.469	-2.79	-4.839	-2.9	1.524	362.722	598.151	533.596	630.937	755.634
139.6	1.201	-1.59	0.109	-0.158	-2.737	513.914	735.594	706.975	757.982	775.851
140.11	-0.693	12.379	-17.829	-6.54	-5.841	526.11	1569.585	311.843	735.416	637.783
140.63	10.529	-7.827	-16.799	-15.546	-5.852	598.429	652.392	722.161	737.643	766.955
141.15	6.52	-8.851	-9.285	-6.456	6.225	542.257	903.373	979.719	751.938	1095.95
141.66	-0.032	7.113	1.011	-3.923	17.108	463.721	1011.619	1241.795	789.138	1301.604
142.18	-0.688	-5.135	-2.011	0.779	4.219	547.211	893.884	997.549	1276.425	1335.505
142.7	23.077	6.917	-5.602	0.696	0.512	666.839	964.229	552.019	1313.084	1536.241
143.21	-24.902	-13.661	-21.524	-20.615	-4.482	456.67	1096.892	1010.538	938.141	1357.788
143.73	25.564	0.184	-1.408	0.297	10.309	882.29	1215.012	1024.113	1587.592	1908.991
144.24	14.885	7.293	5.523	9.797	15.233	566.214	1653.391	826.71	1563.328	1205.909
144.76	-0.366	-2.757	-8.441	-2.343	7.13	465.26	918.973	1107.068	1666.557	1515.213
145.28	9.958	0.379	1.461	7.373	8.832	681.431	1008.161	1605.231	1204.805	1367.851
145.79	16.227	3.099	7.004	7.99	10.386	597.669	998.943	960.131	1080.577	1278.896
146.31	10.278	6.907	6.705	0.285	12.623	634.073	1110.44	1781.642	1023.165	1405.509
146.83	7.917	-1.354	0.024	2.029	11.198	538.573	1692.596	1123.895	1747.164	1331.015
147.34	13.868	9.458	7.45	4.628	14.895	689.133	1154.704	1111.567	1176.576	1291.48
147.86	8.707	9.333	10.465	12.325	11.193	662.222	1019.852	949.336	1116.225	1308.349
148.38	13.86	3.505	4.103	7.825	7.296	713.111	1105.605	1138.904	1116.817	1357.483
148.89	9.85	0.825	4.542	-2.694	6.801	719.262	1129.21	1033.633	1261.431	1324.787
149.41	2.606	3.691	5.53	3.969	11.07	766.192	1112.073	1152.269	1268.952	1321.589
149.92	15.661	8.143	11.028	4.59	12.902	671.611	1092.818	1103.438	1235.115	1414.456

150.44	17.333	1.19	5.786	5.158	9.57	662.767	1149.273	1161.592	1235.77	1421.251
150.96	7.011	6.355	6.798	5.488	13.256	646.368	1154.901	1107.417	1238.73	1338.62
151.47	12.135	1.307	2.995	5.714	9.307	678.994	1218.436	1225.103	1218.045	1336.675
151.99	8.931	3.613	5.284	6.051	14.013	662.582	1173.133	1193.5	1180.372	1408.493
152.51	13.368	8.145	9.184	4.873	9.023	645.75	1201.086	1161.689	1366.854	1362.935
153.02	9.255	3.226	4.544	-0.312	15.521	646.816	1176.53	1172.93	1255.086	1370.049
153.54	7.178	0.679	4.086	1.494	-0.429	643.001	1253.797	1210.082	1170.891	1419.805
154.06	13.046	5.457	7.406	5.539	10.16	627.91	1147.568	1133.981	1137.208	1389.49
154.57	15.85	10.169	4.008	7.872	12.588	676.619	1058.31	1138.99	1180.561	1359.856
155.09	14.526	4.651	5.369	6.266	4.466	625.075	1158.483	1169.965	1235.054	1398.159
155.6	3.738	3.628	4.759	1.464	9.726	621.027	1247.779	1229.02	1234.656	1395.785
156.12	7.127	-0.051	7.282	0.472	10.284	604.685	1300.394	1205.889	1184.869	1361.005
156.64	8.398	10.713	7.189	3.712	11.081	583.053	1254.8	1246.612	1222.761	1387.453
157.16	12.585	5.952	6.163	3.58	6.226	665.646	1288.88	1098.328	1256.707	1358.803
157.67	8.411	6.015	3.28	4.493	9.65	616.253	1110.799	1217.641	1200.484	1403.89
158.19	12.085	6.395	2.107	5.68	10.713	595.824	1152.74	1263.256	1214.113	1447.343
158.71	9.518	3.583	3.944	3.609	8.672	604.537	1170.836	1247.214	1139.364	1440.843
159.22	5.492	2.43	7.382	6.026	10.479	645.062	1326.62	1275.487	1206.285	1432.443
159.74	7.83	-1.246	0.172	2.485	7.905	608.477	1337.031	1278.5	1169.787	1491.516
160.26	6.728	4.362	2.858	-1.517	7.07	654.89	1282.526	1269.741	1202.896	1380.897
160.77	5.574	4.302	3.155	0.969	9.622	544.47	1300.616	1355.272	1256.171	1484.891
161.29	11.939	5.467	4.366	5.366	7.006	599.408	1267.443	1299.14	1240.008	1452.346
161.81	8.379	4.877	2.23	7.148	16.33	568.201	1216.855	1177.486	1174.023	1418.475
162.32	0.811	1.624	0.017	-5.132	6.261	688.82	1303.24	1220.673	1129.503	1401.387

Fig 2. Biodegradation rate of IBP by *R. cerastii* IEGM 1278 in a laboratory bioreactor.

Time, h	0			5			10			15			20		
IBP concentration, mg/L	100	100	100	95.1	98.3	96	52.8	58.9	49.5	39.9	46.8	36.4	30.5	43.4	29.1
DO	201.4			180.4			123.2			122			136		

Fig 3. Biodegradation rate of IBP by native and pre-incubated *R. cerastii* IEGM 1278 cells.

	days	0			6			12			18			24		
IBP concentration, μg/L	abiotic control	100	100	100	99.5	100	100	100	100	100	100	100	100	100	100	100
	biosorption control	100	100	100	100	100	100	100	100	100	100	100	100	100	100	100
	IEGM 1278 native cells	100	100	100	90.2	87.9	93.4	80.1	78.6	87.3	78	73.1	80	53.1	48.7	53.2
	IEGM 1278 pre-incubated cells	100	100	100	66.8	72.3	75.8	36.9	40.6	47.7	28.9	30.7	36	20.8	22.9	28.4
CDW, g/L	n-hexadecane	0.1	0.09	0.11	0.27	0.36	0.31	0.77	0.81	0.75	0.8	0.88	0.83	1.15	1.14	1.02
	n-hexadecane + IBP	0.1	0.13	0.1	0.19	0.31	0.24	0.79	0.74	0.77	0.81	0.79	0.82	1.1	1.18	1.07

	days	30			36			42			48		
IBP concentration, μg/L	abiotic control	100	100	100	100	100	100	100	100	100	100	100	100
	biosorption control	100	99.4	100	100	100	100	100	100	100	100	99.6	100
	IEGM 1278 native cells	34.3	36.4	38	24.2	21.4	27.3	6.8	2.9	5.7	0	0	0
	IEGM 1278 pre-incubated cells	0	0	1.8	0	0	0	0	0	0	0	0	0
CDW, g/L	n-hexadecane	1.18	1.17	1.26	1.19	1.24	1.32	1.32	1.41	1.46	1.21	1.32	1.3[illegible]
	n-hexadecane + IBP	1.08	1.18	1.24	1.18	1.29	1.24	1.24	1.44	1.39	1.23	1.39	1.3[illegible]

Fig 8. Correlation of membrane permeability with zeta potential of *R. cerastii* IEGM 1278.

No	0.1% *n*-hexadecane		100 mg/L IBP and 0.1% *n*-hexadecane	
	zeta potential	membrane permeability	zeta potential	membrane permeability
0	-25.1	80.5	-25.15	80.5
1	-25.3	80.65	-25.35	85.6
2	-25.6	82.3	-29.38	70.5
3	-24.7	81.67	-30.4	69.6
4	-25.5	81.36	-35.3	51.3
5	-23.8	82	-36.54	48.6
6	-24.9	81.7	-36.9	46.3
7	-25.7	82.6	-37.5	41.9
8	-25.5	83.7	-37.8	42.5

Table 2. Percentage of IBP (100 mg/L) remaining during biodegradation experiment after 7 days of incubation of the actinobacterial strains in the RS medium.

Strain	*Agromyces mediolanus* IEGM 860	*Corynebacterium variabile* IEGM 824	*Dermacoccus nishinomiyaensis* IEGM 393	*Dietzia maris* IEGM 297	*D. maris* IEGM 302	*D. maris* IEGM 459	*Gordonia terrae* IEGM 153	*Nocardioides albus* IEGM 820	*N. jensenii* IEGM 821	*Rhodococcus cerastii* IEGM 1278	*R. cercidiphylli* IEGM 1184	*R. erythropolis* IEGM 501	*R. erythropolis* IEGM 711	*R. fascians* IEGM 1158	*R. ruber* IEGM 596	*R. ruber* IEGM 477
1	96.5	97.3	100	93.41	92.23	89.34	93.21	90.75	88.75	86.29	77.94	81.01	95.46	97.38	87.41	94.45
2	96.9	98.44	100	91.1	95.42	94.95	95.9	88.16	87.52	87.89	76.91	80.69	95.17	99.34	88.53	98.86
3	95.4	96.2	100	95.37	88.55	83.7	90.49	92.07	90.28	83.6	80.27	82.51	95.39	96.91	92.48	93.6
Mean	96.3	97.3	100.0	93.3	92.1	89.3	93.2	90.3	88.9	85.9	78.4	81.4	95.3	97.9	89.5	95.6
SD	0.78	1.12	0.00	2.14	3.44	5.63	2.71	2.10	1.38	2.17	1.72	0.97	0.15	1.30	2.67	2.82

Table 3. **Experimental phytotoxicity of IBP and its biotransformation products.**

No	IBP biotransformation products, 1/1.000			IBP biotransformation products, 1/100			IBP biotransformation products, 1/10			IBP biotransformation products			Control (water)	IBP 100 mg/L
1	100	40	120	134	155	128	75	145	55	115	98	47	170	45
2	143	115	84	142	179	103	95	147	126	75	43	60	62	116
3	100	130	85	133	96	116	81	122	98	80	42	62	72	42
4	120	147	134	153	115	95	103	65	117	86	59	70	120	35
5	120	164	61	68	108	99	153	75	102	59	55	50	152	56
6	120	113	131	153	100	85	105	121	61	60	56	50	108	80
7	116	50	142	129	140	73	86	91	124	42	70	40	161	41
8	156	137	131	82	119	89	74	93	91	64	49	46	127	98
9	115	118	101	92	132	116	54	94	116	55	56	59	142	91
10	90	113	110	112	116	125	93	50	74	75	77	32	154	112
11	120	52	56	132	114	129	114	112	119	60	40	55	120	115
12	140	135	119	87	120	164	108	157	137	68	52	70	53	47

13	124	82	90	93	119	137	75	76	133	71	72	64	133	89
14	85	89	96	140	71	86	45	123	82	85	80	86	144	44
15	112	142	140	122	97	105	108	45	80	75	79	70	141	110
16	93	117	93	111	76	88	120	120	93	35	96	70	124	78
17	140	100	121	156	120	86	110	165	140	60	82	55	125	79
18	110	149	151	122	133	145	100	94	102	68	86	81	102	50
19	90	124	119	53	98	110	97	141	147	84	38	61	153	73
20	64	169	103	140	62	155	55	137	108	75	45	40	74	76
21	157	100	112	138	134	96	120	61	121	78	75	50	114	98
22	121	90	66	142	92	124	60	102	106	40	66	70	135	84
23	96	74	156	111	101	130	72	80	110	40	70	85	118	38
24	125	95	149	110	80	121	76	90	82	63	55	64	114	91
25	84	128	81	141	95	108	46	72	57	71	75	52	113	115
Mean / SD	111.5 / 1.87			114.4 / 4.73			98.5 / 8.29			63.9 / 4.04			121.2 / 2.32	76.1 / 3.11

Table 7. Morphometric parameters of *R. cerastii* IEGM 1278 cells grown in the RS medium supplemented with IBP and *n*-hexadecane.

No	0.1 % *n*-hexadecane						100 mg/L IBP + 0.1 % *n*-hexadecane					
	1 day			4 days			1 day			4 days		
	Length, µm	Width, µm	Roughness, nm	Length, µm	Width, µm	Roughness, nm	Length, µm	Width, µm	Roughness, nm	Length, µm	Width, µm	Roughness, nm
1	3.7	1.1	135.9	3.2	0.8	120.0	3.0	1.1	210.4	2.1	1.0	100.7
2	3.8	0.9	134.7	4.1	1.1	177.2	2.9	1.0	171.7	4.1	0.8	100.0
3	3.6	0.8	111.2	2.9	0.8	130.8	3.4	1.2	157.1	2.4	0.9	130.2
4	3.9	1.0	140.2	3.6	0.4	166.0	3.3	1.1	120.5	2.3	1.1	110.9
5	3.8	1.1	144.5	3.7	0.5	110.0	3.2	1.1	139.0	2.9	0.9	109.9
6	3.9	1.2	158.8	3.9	0.6	119.5	3.1	1.2	170.1	2.0	1.1	210.7
7	4.4	1.2	121.9	3.7	0.6	135.0	2.8	0.9	116.9	1.9	0.7	103.5
8	3.9	1.1	119.7	4.1	0.5	204.6	2.7	1.0	180.6	2.2	0.9	110.0
9	3.7	1.0	116.8	3.3	0.5	129.0	3.2	1.2	144.2	3.0	1.1	106.9
10	3.8	0.9	113.6	3.5	1.0	119.6	3.3	1.0	175.9	2.4	1.0	100.7
11	4.0	1.2	100.3	3.0	0.6	124.0	3.1	1.1	184.6	2.3	0.7	106.7
12	3.4	1.0	120.9	2.9	0.5	125.7	3.2	1.1	168.5	2.5	0.8	220.7
13	4.3	1.0	159.1	3.4	0.6	118.9	3.0	1.0	210.9	2.0	1.0	104.1
14	3.7	1.0	112.3	3.9	0.9	133.1	3.1	1.0	179.0	2.3	1.0	103.4
14	4.2	1.0	130.8	3.0	0.7	164.9	3.4	0.9	176.7	2.1	0.9	105.7
16	4.2	1.2	127.3	3.5	0.5	120.9	3.3	1.1	158.9	1.9	1.1	100.5
17	3.7	1.2	126.1	3.0	0.7	123.7	3.4	1.0	198.0	3.0	0.8	100.0
18	3.7	0.8	124.7	3.9	0.7	124.8	3.2	1.1	140.9	2.1	0.8	101.4
19	3.9	1.0	118.0	4.0	1.0	134.0	3.1	1.0	175.0	2.6	0.8	105.9
20	3.7	1.0	134.1	3.8	0.5	129.8	3.5	0.9	158.9	2.0	0.9	163.0
21	3.5	0.8	140.3	4.0	0.9	99.0	3.6	1.0	208.6	2.5	1.1	100.4
22	4.1	1.2	156.8	3.5	0.8	132.0	3.2	1.1	163.0	2.0	1.1	115.7
23	4.2	1.2	131.1	3.3	0.4	160.5	2.8	1.0	123.5	2.2	0.9	100.0
24	3.7	1.1	152.7	3.1	0.5	136.8	3.2	1.2	163.9	3.1	1.1	104.7
25	3.8	1.1	141.8	4.1	0.5	123.0	3.0	1.1	160.3	2.0	1.1	155.8
26	3.1	1.0	158.1	2.8	0.4	113.7	3.4	1.1	189.0	2.5	1.0	212.0
27	4.2	1.0	166.9	3.4	0.5	125.2	3.2	1.2	211.0	2.1	0.9	100.0

28	3.8	0.8	171.9	3.7	0.5	125.1	3.1	1.0	175.6	4.0	1.1	101.3
29	3.9	1.1	112.7	3.6	0.4	134.8	3.3	1.0	168.4	1.9	1.0	100.8
30	3.6	0.8	120.8	2.8	1.0	225.4	3.0	1.1	185.7	3.0	0.9	160.9
Mean	3.8	1.0	133.5	3.5	0.6	136.2	3.2	1.1	169.6	2.4	1.0	121.6
SD	0.28	0.14	18.49	0.42	0.21	27.33	0.21	0.09	25.54	0.57	0.13	36.17
